# Distributions of Autocorrelated First-Order Kinetic Outcomes: Illness Severity

**DOI:** 10.1371/journal.pone.0129042

**Published:** 2015-06-10

**Authors:** James D. Englehardt

**Affiliations:** Civil, Architectural, and Environmental Engineering, University of Miami, Coral Gables, Florida, United States of America; University of Zurich, SWITZERLAND

## Abstract

Many complex systems produce outcomes having recurring, power law-like distributions over wide ranges. However, the form necessarily breaks down at extremes, whereas the Weibull distribution has been demonstrated over the full observed range. Here the Weibull distribution is derived as the asymptotic distribution of generalized first-order kinetic processes, with convergence driven by autocorrelation, and entropy maximization subject to finite positive mean, of the incremental compounding rates. Process increments represent multiplicative causes. In particular, illness severities are modeled as such, occurring in proportion to products of, e.g., chronic toxicant fractions passed by organs along a pathway, or rates of interacting oncogenic mutations. The Weibull form is also argued theoretically and by simulation to be robust to the onset of saturation kinetics. The Weibull exponential parameter is shown to indicate the number and widths of the first-order compounding increments, the extent of rate autocorrelation, and the degree to which process increments are distributed exponential. In contrast with the Gaussian result in linear independent systems, the form is driven not by independence and multiplicity of process increments, but by increment autocorrelation and entropy. In some physical systems the form may be attracting, due to multiplicative evolution of outcome magnitudes towards extreme values potentially much larger and smaller than control mechanisms can contain. The Weibull distribution is demonstrated in preference to the lognormal and Pareto I for illness severities versus (a) toxicokinetic models, (b) biologically-based network models, (c) scholastic and psychological test score data for children with prenatal mercury exposure, and (d) time-to-tumor data of the ED_01_ study.

## Introduction

Regulation of chronic chemical toxicants generally involves the use of a quantal dose—response function, i.e. a relationship describing the fraction of a population (human or animal) expected to become ill as a function of the level of exposure to, or dose of, a stressor (often a chemical) after a certain exposure time. The function may be interpreted alternatively as the probability that a randomly-selected individual will become ill, as a function of dose. In either case, the positive illness condition corresponds to a level of clinical diagnosis, such as pulmonary function, that may be termed illness severity.

Toxicant regulation often implies the extrapolation of a quantal dose-response function over orders of magnitude, from high doses at which effects can be observed in small laboratory populations, to low doses of regulatory interest for which e.g. only one illness may occur in 100,000 individuals. Results of the extrapolation may depend critically on the form of the dose-response function (DRF) [1], pointing to the need for a theoretical basis. Mathematically, the form of the DRF is determined by the form of the illness severity distributions, i.e. the set of functions representing population fraction presenting, versus a quantitative representation of a clinical diagnosis, at the corresponding set of fixed doses. Hence, a theoretical basis for the form of the illness severity distribution would provide basis for the form of the DRF. Fortunately, illness severity is the magnitude of an outcome of a complex physiological system, and distributions of complex system outcome magnitudes have shown commonalities across fields.

### Distributions of illness severity

The form of the distribution of illness severities, across a population at fixed dose, has not been well-studied. For convenience in analysis of dose-response data, distributions of the severity of mild effects, such as changes in body weight, are sometimes assumed approximately normal or lognormal [2]. However, of these, only the lognormal distribution is theoretically appropriate in terms of its range over the positive real line.

Illness severity distributions may be inferred from certain biomarker data, (i.e., from a measurable indicator of the severity of an illness condition), such as toxicant concentration in arterial blood. For example, Clewell and coworkers [3] modeled steady-state (lifetime) arterial blood concentrations for oral exposure to volatile compounds as:

$$C_{art}=\frac{Drink*Q_{1}}{Cl_{1}*V_{a}/H_{ba}}$$ (1)

in which *Drink* is a continuous zero-order ingestion rate, *Q*_*l*_ is blood flow to the liver, *H*_*ba*_ is the styrene blood:air partition coefficient, *V*_*a*_ is the minute ventilation rate, and *Cl*_*l*_ is the metabolic clearance of styrene in the liver. If it were possible to extend the approach of Eq 1 to obtain a distribution of illness severities, by accounting for subsequent pharmacodynamic actions, a similarly multiplicative relationship might result.

Several of the variables in Eq 1 might potentially be modeled with lognormal distributions [4]. In that case, because reciprocals of lognormal RVs are lognormal, the product would be a lognormal-like distribution of arterial blood concentration if the variables were either largely independent (by the central limit theorem), or perfectly correlated (by transformation of powers of lognormal variates) [5]. However, the lognormal necessarily predicts zero probability of zero severity. In contrast, the distribution of illness severities across a population is often monotonically-decreasing, i.e. highest probability associated with no illness, particularly for a low dose and/or a serious endpoint. For example, for many important endpoints, e.g. carcinoma, hypertension, the most prevalent condition is no illness, e.g. normally functioning (or wild type) genes or normal blood pressure.

### Distributions of complex system outcomes

In other fields, the form of the distributions of complex system outcomes has been widely observed to be decidedly non-Gaussian, nearly log-log linear, or power law, in shape over orders of magnitude in range [6–10]. If truly log-log linear, such distributions would “scale,” or remain invariant in form with respect to change in scale, a property they would share with many other continuous and infinite/semi-infinite probability distributions; therefore such distributions have been referred to as *scaling* or *scale-free*. However, the observed distributions often have curvature. More important, the log-log linear form must break down at one or both extremes in order to maintain normalization. As a result, exponential cut-offs for power laws have been used to fit datasets empirically. However, data are needed to position and fit the cutoff function, generally precluding extrapolation. Therefore, a more general single function, capable of capturing the underlying emergent mechanisms over a broader range, would be needed e.g. for regulatory dose-response extrapolation.

Although scaling distributions have been associated with systems exhibiting self-similarity (fractal patterns) [7], self-organized criticality (cascading failures punctuating near-equilibrium conditions) [9], preferential attachment (e.g., the tendency for new citations to cite previously-cited work, and web sites to link to more highly-linked sites) [6,10], and other mechanisms, no unified explanation for their recurrence has emerged. One explanation has been that such distributions arise as normed sums of independent, identically-distributed random variables (RVs) having infinite variance [11], stable by the Central Limit Theorem though non-Gaussian. Stable distributions are attracting, i.e. convergent with continued summation of normed independent and identically-distributed random variables. However, an infinite variance may not be realistic over any range.

In 1951 Weibull [12] demonstrated the “wide applicability” of his namesake distribution to disparate complex system outcomes, including strength properties of materials; fly ash particle size; *Cyrtoideae* length; *Phaseolus vulgaris* bean width; and heights of adult males born in the British Isles. Much later, Mittnik and Rachev showed the Weibull distribution to dominate stable distributions in terms of fit to S&P 500 stock return data, as explained by a proposed stability of minimum values, and stability of sums of geometrically-distributed numbers of RVs [13]. Sornette and coworkers then demonstrated the Weibull (referred to as the stretched exponential) distribution versus a wide array of observed heavy-tailed distributions [14], including of radio and light emissions from galaxies; US oil field reserve sizes; global, US and French urban agglomeration sizes; national population sizes; daily Forex US-Mark and Franc-Mark price variations; Vostok temperature variations over 400,000 years; Raup-Sepkoski's kill curve; and citations of highly cited physicists.

### Applicability of the first-order kinetic model

Sornette and coworkers explained the generality of the Weibull distribution by suggesting that complex system outcome magnitudes emerge as products of the magnitudes of a series of independent outcome causes, and showing that products of independent elements having certain exponential-family distributions are asymptotically Weibull-distributed in the tail [15], contrasting with the lognormal result indicated by the Central Limit Theorem. Subsequently, we proposed complex process outcomes to arise rather generally as products of cause magnitudes that are instead autocorrelated, and argued the multiplicative causal elements to be exponentially-distributed by entropy-forcing [16], as will be described. We later presented arguments that products of discrete autocorrelated causes have the discrete Weibull distribution, as demonstrated versus counts of *C*. *parvum*, *Giardia*, total coliform, fecal coliform, and fecal streptococci in water [17]. Significantly, we also found the autocorrelated multiplicative model to produce 1/*f* spectra (a power law relationship of amplitude versus frequency in time series data, with slope approximately unity), another characteristic of many complex systems, when the outcome magnitudes themselves are autocorrelated [18].

Mathematically the multiplicative model is equivalent to a first-order kinetic process [19], as discussed later under Review of Model Bases. Further, many biological, chemical, and economic quantities grow or decay by first-order kinetics, such that their change in size is proportional to their current size [20]. The relationship is ubiquitous because many changes in magnitude occur largely in proportion to the availability of a single limiting monetary, human, animal, plant, microbial, or chemical species. Hence the first-order, or multiplicative, model may represent a rather general explanation for the occurrence of power law-like distributions, at many scales. In particular, chronic toxicants are generally present in low concentrations, such that reaction saturation effects are unimportant. For example, the effect of a toxicant may occur in proportion to its concentration, while concentrations of other reactants remain in relative excess or otherwise constant and non-limiting. At a larger scale, the total fraction of a toxicant passed to a target organ is similarly proportional to the fractions passed by preceding organs. Such pseudo-first-order reactions have rate constants which vary along a toxicological pathway, for example between organs and in time (as well as among individuals), and these variations are generally autocorrelated along the pathway as discussed under Review of Model Bases.

### Objectives

The purpose of this paper is to derive and demonstrate the form of the distribution of the outcomes of autocorrelated first-order kinetic processes, with application to toxicological pathways. First, the first-order model is written as a serial multiplication of incremental terms, which are generally variable and autocorrelated in size. The increments along a toxicological pathway, in particular, are viewed as elementary causes of illness, and the information-theoretic basis for their distributions is reviewed. The asymptotic form of the illness severity distribution is then derived, and shown to converge as a result of autocorrelation across increments. To maintain generality, the result is demonstrated versus general kinetic, toxico-kinetic, and network models of pathogenesis, and extended to the case of saturation kinetics. Results are then demonstrated versus published data on illness severity, including data on learning disabilities in children with prenatal mercury exposure, and transformed time-to-tumor data.

## Methods

Some terms are defined as follows. First-order refers to a generalized abiotic or biotic kinetic process in which growth or decay occurs predominantly in proportion to current magnitude, via single, parallel and/or network pathways, i.e. with overall rate law approaching d*M*/d*t* = ±*rM*, in which *M* is the magnitude at time, *t*, and *r* is a rate constant. The size of a cause of an illness or other incident is its magnitude, for example the fraction of toxicant passed to a receptor (not eliminated), or more generally the degree of failure of a protective system. The more general terms first-order process increment, and process increment, are used interchangeably with cause. Elementary causes are initial causes, not related in size to other causes. Autocorrelation refers to positive serial dependence along a sequence of RVs as defined by a positive Pearson correlation coefficient, and perfect correlation among a set of *J* random variables is defined by a *J* x *J* correlation matrix in which all elements are equal to 1. Entropy refers to classical Shannon entropy [21]. The notations f(.) and F(.) denote continuous probability density function (PDF) and cumulative distribution function (CDF), respectively. The term standard exponential distribution is used to indicate an exponential distribution with a mean of unity.

Distributions of autocorrelated first-order outcomes were simulated using Matlab version R2006a and R2010a with Statistics Toolbox. Each vector of *J* partially correlated exponential RVs was simulated by the copula method [22], as a vector of *J* exponential inverse-CDF values for *J* standard normal CDF values corresponding to a *J*-element multivariate normal (**μ**, **ρ**) random vector with mean **μ** = **0** and covariance matrix **ρ** equal to a *J* x *J* subset of the matrix below:

- [1 0.66 0.56 0.46 0.36 0.26 0.16 0.06 0 0 …
- 0.66 1 0.66 0.56 0.46 0.36 0.26 0.16 0.06 0 0 …
- 0.56 0.66 1 0.66 0.56 0.46 0.36 0.26 0.16 0.06 0 0 …
- 0.46 0.56 0.66 1 0.66 0.56 0.46 0.36 0.26 0.16 0.06 0 0 …
- 0.36 0.46 0.56 0.66 1 0.66 0.56 0.46 0.36 0.26 0.16 0.06 0 0 …
- 0.26 0.36 0.46 0.56 0.66 1 0.66 0.56 0.46 0.36 0.26 0.16 0.06 0 0 …
- 0.16 0.26 0.36 0.46 0.56 0.66 1 0.66 0.56 0.46 0.36 0.26 0.16 0.06 0 0 …
- 0.06 0.16 0.26 0.36 0.46 0.56 0.66 1 0.66 0.56 0.46 0.36 0.26 0.16 0.06 0 0 …
- 0 0.06 0.16 0.26 0.36 0.46 0.56 0.66 1 0.66 0.56 0.46 0.36 0.26 0.16 0.06 0 0 …
- 0 0 0.06 0.16 0.26 0.36 0.46 0.56 0.66 1 0.66 0.56 0.46 0.36 0.26 0.16 0.06 0 0 …
- … 0 0 0.06 0.16 0.26 0.36 0.46 0.56 0.66 1 0.66 0.56 0.46 0.36 0.26 0.16 0.06 0 0 …
- …
- …
- …]

This procedure produced a maximum correlation between (adjacent) exponential causes of 0.61. Generic metabolic networks were modeled using Pajek 1.20 network analysis software.

Simulated PDFs were fitted with distributions by maximum likelihood estimation (MLE). Simulated CDFs were fitted by goodness-of-fit (GOF) maximization, i.e. by minimizing the deviance statistic, an adaptation of the 2-log likelihood ratio to quantal data [23]. Model fits were evaluated by graphical comparison with empirical densities (*N* = 1000). To avoid possible shape distortion associated with the assumption of arbitrary bin widths, empirical densities were obtained by assigning each unique observed value of outcome size its own histogram bin, as described previously [16,24,25]. That is, the probability density for each bin was found as the fraction of outcomes having that size (that is, *m*/*M*, in which *m* is the number of observations of that outcome size, typically one for real-valued sizes, and *M* is the total number of observations), divided by the bin width. Bin bounds were determined as follows. First, for discrete data over the range 0, 1, 2, …, unit-width bins must begin at the count value (e.g., zero) and extend up to, but not include, the next count value (e.g., one). This observation is consistent, e.g., with the observation that viable microbe counts are effectively rounded down to exclude fractions of microbes. For continuous data, the probability of measuring exactly zero is zero. Therefore, again data are effectively left-censored at the limit of measurement precision, even if zero-valued data are recorded as a result of rounding. Therefore, bin widths were measured from the data point up to, but not including, the next observed value. Accordingly, the last point served only to define the upper limit of the last bin, given the observed record length. The density was then plotted at the data point. This approach maximizes the number of bins and minimizes the effect of the unknown upper limit of the final bin, particularly influential for heavy-tailed data. Such empirical PDFs are maximally objective, though entirely unsmoothed so that even large samples have wide-ranging residuals.

The chi squared and asymptotically-equivalent [26] log-likelihood ratio GOF tests were used for their ability to indicate the probability with which a tested distribution can be rejected, correcting the degrees of freedom for the number of distribution parameters. However, even these tests will consistently reject the true distribution for very large datasets. For example in this work, values of *α* = 0.05 and *N* = 1000 to 10,000, depending on parameter values, were found to accept the true sampling distribution of simulated Weibull-distributed RVs 94 out of 100 times, but to consistently reject the true distribution for larger data sets (*α* = 0.05). Therefore, model output was subjected to chi-square testing, at *N* = 1000, repeated 100 times and reported as the number of records passing at *α* = 0.05. GOF tests were coded in Matlab version R2006a and R2010a with Statistics Toolbox software.

Published quantal time-to-tumor data were fitted and plotted using the plotting position Min[Max(0.25, *m*/*M*), *M*-0.25], in which *m* is the number of illness-positive individuals (e.g., mice) and *M* is the total number of individuals. That is, a data point of *m* = 0 positives suggests a population illness proportion of from 0/*M* to 0.5/*M*, with a midpoint of 0.25/*M*. If the data point is interpreted as the extreme value, 0/*M*, that point cannot be used to assess the log-likelihood (because the result is undefined), whereas use of the interval midpoint allows use of the point. Likewise, if *m* = *M* is observed and the population proportion is interpreted at the interval extreme of *M*/*M*, the point cannot be used. Thus it was assumed for example that responses of zero of 10 mice, and 10 of 10 mice, would have been proportional to responses of 0.25 in 10, and 9.75 of 10, respectively, if sample sizes had been larger. This plotting position is consistent with others published [27], and was used to better approximate the mean binomial probability of response at times for which all or no mice were positive, and to allow GOF fitting and visual assessment at these important times. Background risk was corrected for by subtracting the proportion of positive mice at zero dose, times the total number of mice tested at each nonzero dose, from the number of positive mice at each nonzero dose. In cases where no positive mice were observed for the first several observation times, only the last two of those times were considered in the analysis, on the assumption that population probabilities at even lower doses were actually zero (doses below toxic threshold) and therefore could not be used in the log-likelihood analysis, consistent with standard practice. Data were tested for GOF by evaluation of the deviance statistic versus the chi-squared distribution.

## Review of Model Bases

In this section, bases for the autocorrelated first-order model, including its multiplicative nature, and the autocorrelated and exponentially-distributed character of the causal elements, are described.

### Multiplicative and autocorrelated character of first-order processes

A first-order kinetic model, such as one of decomposition in a single organ, is a multiplicative process equivalent to the multiplication of cause magnitudes along a causal chain such as a toxicological pathway. For discrete growth/decay the equivalent multiplicative relationship is well-known:

$$\begin{array}{l} \frac{\Delta A}{\Delta t}=rA\\ A_{T}\;=A_{0}\prod_{t=1}^{T}\left(1+r\right) \end{array}$$ (2)

in which *A* is e.g. the concentration of toxicant A, *T* is the total period of reaction time, *t*, or distance along a pathway, II indicates a product, and the second equation is equivalent to *A*_*T*_ = *A*_*0*_(1+*r*)^*T*^. While the real rate constant for growth/decay, *r*, varies in general as -1 < *r* < ∞ (because *A* cannot decay to values of zero or less, nor grow at an infinite rate), the term (1+*r)* is positive.

Though often assumed constant, the term (1+*r*) generally varies randomly in a Markovian manner (i.e., future value is determined solely by present value, independent of past values, such that long-term memory is lost) among individuals and in time, potentially assuming values less than and greater than unity among time increments, and is thus generally autocorrelated along the kinetic pathway. For example, monetary interest rates, and toxicant decay rates within an organ, are correlated in time, and elimination and transformation rates are correlated along a multi-organ toxicological pathway, as will be explained. In the latter case, toxicant concentration at the target organ is equal to the product of the fractions passed by preceding organs:

$$\begin{array}{l} A_{T}\;=A_{0}\prod_{t=1}^{T}\left(1+r_{t}\right)\\ \;=A_{0}\prod_{t=1}^{T}f_{t} \end{array}$$ (3)

in which *f*_*t*_ = *A*_*t*_ */A*_*t-1*_ is the fraction of toxicant, or metabolic product thereof, passed at a pathway increment. Thus, a first-order kinetic model is equivalent to a multiplicative model that could be invoked to describe, for example, multi-organ transfer of toxicant, or the product of probabilities (rates) that mutations in oncogenic or tumor suppression genes escape cell growth controls and correction processes such as DNA repair mechanisms or apoptosis.

While not generally thought of as such, continuous growth/decay, d*A*/d*t* = *rA* and *A* = *A*_*0*_e^*rt*^, is viewed here as a continuous serial multiplication, a special case of Eq 2 as Δ*t* → 0, as follows:

$$\begin{array}{l} A_{T}=\begin{array}{c} \text{lim}\\ \Delta \text{t}\to \text{0} \end{array}\;A_{0}\prod_{t}^{T}\frac{A_{t}}{A_{t-1}}\\ \;=\begin{array}{c} \text{lim}\\ \Delta t\to 0 \end{array}\;A_{0}\prod_{t}^{T}\frac{A_{0}{\left(1+r\right)}^{t}}{A_{0}{\left(1+r\right)}^{\left(t-\Delta t\right)}}\\ \;=A_{0}\pi_{0}^{T}\frac{A_{0}e^{rt}}{A_{0}e^{r(t-dt)}}\\ \;=A_{0}\pi_{0}^{T}e^{rdt} \end{array}$$ (4)

in which

$$\begin{array}{l} \pi_{a}^{b}{\left[f(x)\right]}^{dx}=\begin{array}{c} \text{lim}\\ \Delta x\to 0 \end{array}\prod {\left[f(x_{i})\right]}^{\Delta x}\\ \;=\text{exp}\int_{a}^{b}\text{ln}f(x)dx \end{array}$$ (5)

is a continuous product, or multiplicative analog to the Riemann integral, known as a product integral [28], and the non-negative, real term e^*r*^ is analogous to the term (1+*r*) of the discrete case. In other words, a continuous first-order kinetic process can be viewed as a continuous multiplication of continuously varying random, vanishingly small incremental cause sizes (though the outcome size may be real or integer). In this paper, the quantities e^*r*^ and (1+*r*) are referred to as *rate multipliers*, for continuous and discrete models, respectively.

Although use of the product integral in Eqs 4 and 5 represents an unfamiliar representation of a known result, it is fundamental to developing the continuously multiplicative nature of the continuous first-order kinetic rate law, not otherwise obvious. In particular, it indicates that if Δ*t* → d*t* < 1, then outcomes can arise as the product of several fractional compounding increments that collectively represent less than one cause or compounding period. Thus, Eqs 4 and 5 provide the required basis for simulation of such “continuous” first-order processes in the Results section. Further, they clarify the definition of a population geometric mean [16].

As previously discussed, first-order kinetics are ubiquitous, suggesting applicability across scales in many processes. For example, at a toxicological pathway scale, illness severity may be modeled generally as $Z\;=C_{1}\prod_{j=1}^{J}C_{j}$, in which *Z* is illness severity; *C*_*1*_ is the dose; subsequent *C*_*j*_ are the *j*-th succeeding random cause sizes corresponding to rate multipliers, (1+*r*) or e^*r*^; and *J* = *T* is the number of discrete causes or, equivalently, the period of continuous first-order compounding. The result of such multiplicative processes is that causes or initial process outcomes that are either small or large become disproportionately more extreme as the process continues. If the rate constant of the process varies randomly along its path, and/or with the size of the first cause, *C*_*1*_, then the size of the outcome over many trials is described by a probability distribution.

Increments of first-order processes, or causes, are generally highly inter-related and positively autocorrelated in size [16,29], for several reasons [16]. First, “common-mode” failures occur in engineered systems, e.g. when multiple components are made from the same batch of defective material or calibrated with a common faulty instrument. In such cases, the simultaneous failure of the corresponding redundant protective mechanisms may result in a gas explosion, fire, or other negative outcome. Likewise, illness may arise in biological systems when multiple organs are affected by a toxicant or stressor in the circulatory system, when immuno-compromised individuals have generally weakened systems, or when large chromosomal regions are lost. For example, extensive mutation in DNA repair genes may compound the effect of preceding extensive errors in proto-oncogenes and tumor suppressor genes caused by the same critical exposure, allowing a general dysfunction of key genes involved in cell cycle regulation and growth control (and thus a neoplastic state). A second important, distinct class of common mode failure, characteristic of complex systems, is “control-system” failure, e.g. failure of a central processor or circulatory system causing proportional systemic failures.

A third reason for correlation among cause magnitudes is that when “upstream” protective mechanisms are overwhelmed, they may fail more completely, potentially overwhelming subsequent protective mechanisms, such that first-order assimilative rate constants tend low. For example, heavy carbon tetrachloride exposure may overwhelm induced transformation and conjugation enzymes and transport proteins in the liver, compromising clearance and thus overwhelming subsequent metabolic processes, ultimately increasing liver pathology. Likewise, if an upstream valve fails completely in blocking the flow of an explosive gas, a downstream pipe or tank wall may also fail completely. At the other extreme, if initial detoxifying mechanisms in a physiological system easily address the insult, subsequent stages also succeed, such that rate constants tend high. Thus, autocorrelation along a first-order kinetic pathway extends the extremes of the outcome size distribution further, increasing scaling. In fact, metabolic networks have been shown to have scale-free distributions of the numbers of connections per node (reaction site), explained by the model of preferential attachment [6,10].

### Form of cause magnitude distributions

To derive the form of the distribution of the product of random cause magnitudes, the general form of the distribution of the individual cause magnitudes can be considered. One non-negative candidate might be the lognormal, with illness severity then modeled as a product of such lognormal cause sizes. However, although such a first-order model would be fundamentally different from the steady-state concentration model of Eq 1, the final illness severity would similarly be a product of lognormal RVs, resulting in the same non-monotonic, potentially-unrealistic, lognormal distribution of severities.

In general, elementary cause magnitude distributions, like illness severity distributions, are expected to be monotonic, because the most common condition should be no failure of a protective mechanism. For example, while blood pressure as a cause of illness may have an underlying lognormal-like distribution, all values below a clinical threshold are considered normal, therefore not representing a cause of the illness to be diagnosed, such as heart disease. Thus, the distribution of cause size is monotonic, and ranges from the clinical definition of hypertension (e.g. 120/80). In fact, monotonic cause sizes were observed in numerical models of self-organized critical pathogenesis [30].

Consistent with monotonicity, elementary cause magnitude distributions have been assumed [14] and reasoned [16] to be exponential in form. The reason is information theoretic. That is, as the number of e.g. biochemical and metabolic variables across a population increases, the number and range of possible outcomes increase multiplicatively. Then, entropy is the average log of the inverse height of the density and so, assuming normalization, is a measure of distribution “breadth.” Therefore, maximizing entropy is roughly equivalent to maximizing the range of feasible outcomes and, all-the-more-so, the number of ways to satisfy the constraints. The maximum entropy distribution is then realized because it can be obtained in overwhelmingly more ways than any other distribution meeting the constraints. Thus, given the average strength of a protective homeostatic mechanism across a population, e.g. the average fraction of toxicant removed by the liver, and no further general constraints on the entropy of its distribution, this strength will have the exponential distribution [21] as the most likely, or maximum entropy, distribution.

As a note, gas molecular energies have one principal constraint on the entropy of their distribution, that being their mean energy indicated by gas temperature, and in fact such energies have an exponential-form (Boltzmann) distribution. Thus, a mean cause magnitude (e.g., extent of protective mechanism failure) can be viewed as analogous to gas temperature. As a counter-example, the distribution of particle locations in a diffusing fluid may have two governing constraints: mean advective velocity and coefficient of diffusion, corresponding to dynamic mean and variance, respectively. From these, the Gaussian diffusion equation can be derived by maximizing entropy. Many other physical laws can be derived similarly [31,32], if the necessary and sufficient physical constraints of the system can be first identified, and then described mathematically.

## Results

In this section, a new derivation of the Weibull distribution of first-order outcomes is presented, building on arguments we presented previously for the discrete Weibull distribution, and the distribution is demonstrated versus simulated first-order outcomes. On these bases, the term *emergent* is defined and applied to the Weibull and Fréchet distributions. The Weibull distribution is then demonstrated versus physiologically-based kinetic and network models, including a model of saturation kinetics, and versus published cancer and non-cancer dose-response data.

### Derivation of the asymptotic distribution of first-order outcomes

When first-order kinetics comprise serial multiplication of perfectly-correlated, exponentially-distributed increments, then outcome is proportional to a power of increment size, and the distribution of outcomes is Weibull-distributed by transformation of variables [5]. Also, while known distributions of products of partially-correlated exponential RVs are not closed-form [33], Frisch and Sornette [15] showed that products of *n* perfectly independent exponential RVs, and others distributed proportional to exp(-*Dx* ^*γ*^) in which *D* is a constant and *γ* is a parameter, are asymptotically Weibull-distributed in the tail, for large *n*. Hence, only the remaining body of the distribution deviates from the Weibull distribution with decreasing autocorrelation. Thus, the question arises as to whether the Weibull distribution might obtain asymptotically for first-order outcomes over (a) a range of autocorrelation and (b) a range of monotonic elementary cause magnitude distributional forms. In that regard, it is not clear how much of, or how closely, the infinite tail is Weibull-distributed for physically-relevant values of *n*, or even on what scale *n* should be counted. More important, these results regarding systems with perfect independence, and perfect correlation, of kinetic increments are silent regarding the behavior of the distribution between these extremes.

The asymptotic distribution of outcomes of partially-autocorrelated first-order processes are derived here by examining the entropy of the distribution, as follows. First, if each cause, *C*_*j*_, of each outcome, *Z*, has finite positive mean, *θ*_*j*_, then each also has finite positive geometric mean, G(*θ*_*j*_). Assuming constant nonnegative and, with no loss of generality, integer numbers of causes, *J*, these geometric mean cause sizes can be written in terms of *Z* as:

$$\text{G}(\theta_{j})={\left(\prod_{j=1}^{J}C_{j}\right)}^{1/J}=Z^{\eta }$$ (6)

in which *η* = 1/*J*, and the expression for G(*θ*_*j*_) is not closed form for the exponential distribution using Eq 5. Then, the mean of *Z* ^*η*^ across all outcome realizations, E[*Z* ^*η*^], in which E[.] is the expectation operator, is also a finite positive constant [17]:

$$\text{E}[Z^{\eta }]=\text{E}[\text{G}(\theta_{j})]=\bar{\text{G}(\theta_{j})}.$$ (7)

For *C*_*j*_ distributed exponential, the mean and the scale parameters of *C*_*j*_ are equal. Hence, a further constraint specifying the scale of the product as a function of the mean geometric mean cause size, is required. In fact, the first-order process engenders such a constraint, as follows:

$$\begin{array}{l} Z=\prod_{j=1}^{J}C_{j}\\ \text{ln}Z=\sum_{j=1}^{J}\text{ln}C_{j}\\ \text{E}\left[\text{ln}Z\right]=\text{E}\left[\sum_{j=1}^{J}\text{ln}C_{j}\right]\\ \text{E}\left[\text{ln}Z\right]=\text{E}\left[J\text{ln}G(\theta_{j})\right]=\bar{J\text{ln}G(\theta_{j})} \end{array}$$ (8)

The last step follows from the expression of a geometric mean as the exponential of the average logarithm [16], and confirms the existence of a relationship between constraints (7) and (8), through the *θ*_*j*_. A parallel development can be written for continuous first-order compounding, replacing sums with integrals as in Eq 5.

In essence, the natural tendency of an elementary cause magnitude distribution towards higher entropy is most generally constrained in terms of its mean magnitude, itself a sort-of physical “temperature” indicating the average magnitude of potential failure or other cause. In first-order systems, this simple constraint on process increment size engenders a constraint expressing the *η*-th moment of process outcome size (also representing the mean geometric-mean cause size), and a further related constraint expressing the mean log system outcome size.

Having identified the operative physical constraints on first-order outcome size, and formulated mathematical constraints (Eqs 7 and 8) representing the physical constraints, though not the relationship between them, it is straightforward to maximize entropy by the method of Jaynes [31] to obtain the following distribution [32]:

$$\begin{array}{l} \text{max }-\int_{0}^{\infty }\text{f}(z)\text{log}[\text{f}(z)]dz\\ S.T.\;\int_{0}^{\infty }\text{f}(z)dz=1\\ \;\int_{0}^{\infty }z^{\eta }\text{f}(z)dz=\bar{G(\theta_{j})},\;0<\eta <\infty ,\;0<\;\bar{G(\theta_{j})}<\infty \\ and\;\int_{0}^{\infty }\text{ln}z\text{f}(z)dz=\bar{J\text{ln}G(\theta_{j})}\;-\infty <\bar{J\text{ln}G(\theta_{j})}<\infty \\ \text{f}(z)=\text{exp}\left\{-\lambda_{0}-\lambda_{1}\;z^{\eta }-\lambda_{2}\text{ ln}z\right\}=\frac{z^{-\lambda_{2}}\text{ exp}(-\lambda_{1}z^{\eta })}{\int_{0}^{\infty }z^{-\lambda_{2}}\text{ exp}(-\lambda_{1}z^{\eta })dz}\\ \text{f}(z)=\frac{\eta \;\lambda_{1}{\left(\lambda_{1}z\right)}^{\lambda_{2}}}{\;\Gamma \left(\frac{\lambda_{2}+1}{\eta }\right)}\text{exp}\left[-{\left(\lambda_{1}z\right)}^{\eta }\right];\;0\leq z<\infty ,\;0<\lambda_{1}<\infty ,\;-1<\lambda_{2}<\infty \end{array}$$ (9)

Eq 9 is the generalized gamma distribution, a limiting distribution subject to the constraints shown, when the three constraints are independent, in which *λ*_0_, *λ*_1_, and *λ*_2_ are Lagrange multipliers (constants); *η* is a shape parameter equal to 1/*J* for discrete compounding [for continuous compounding, 1/(*J*Δ*t*), as will be shown in the next section]; *λ*_*1*_ is a scale parameter; and *λ*_*2*_ is a shape parameter.

The third constraint of Eq 9 can be seen related to the correlation among the *C*_*j*_. That is, if the *C*_*j*_ are correlated, then when the *C*_*j*_ tend higher than unity, their product will tend higher still as they become disproportionally more extreme, such that ln[*Z*] will tend high, and vice versa when the *C*_*j*_ tend low. When the *C*_*j*_ are not correlated, the effect will be reduced, and E[ln(*Z*)] will tend closer to zero. Thus, the absolute value of E[ln(*Z*)] tends toward a direct relationship with correlation.

When first-order increments (cause sizes) are perfectly exponential and autocorrelated, the Weibull form obtains, in which case the previously noted relationship between the last two constraints of Eq 9 is *λ*_2_ = *η*- 1:

$$\text{f}(z)=\frac{\eta }{\xi }{\left(\frac{z}{\xi }\right)}^{\eta -1}\text{exp}\left[-{\left(\frac{z}{\xi }\right)}^{\eta }\right];\;0<\xi ,\;\eta ,\;0\leq z<\infty$$ (10)

The facts that the Weibull special case of Eq 9 (a) obtains exactly for the perfectly-correlated special case of the physical constraints proposed, and (b) has parameters that are related generally as predicted, suggest that the proposed constraints are both necessary and sufficient. (For other systems: without the second constraint of Eq 9 the result would be a power law regardless of cause size distribution [34]; if *η* = *J* = 1, i.e. no first-order growth/decay, the gamma is obtained [32]). However, when first-order increments are partially correlated, the distribution is not well-characterized.

To see how first-order increment autocorrelation affects the shape and resulting entropy of Eq 9, note that whenever *J* (for continuous compounding, *J*Δ*t*, as shown in the next section) is fixed, *η* is constant, and when the increment scales, *θ*_*j*_, are fixed, the scale (units) of the product of the increments, *λ*_*1*_ in Eq 9, must also be constant. Therefore, *λ*_*2*_ of Eq 9 must account completely for the degree to which first-order increments are exponential and autocorrelated. Furthermore, using the general expression for the entropy of a maximum entropy distribution [35], the entropy of Eq 9 is:

$$H_{\text{max}}=\lambda_{0}+\lambda_{1}E[Z^{\eta }]+\lambda_{2}E[\text{ln}Z]$$ (11)

Thus, as process autocorrelation varies, ∂*H*_*max*_/∂*λ*_*2*_ = E[ln(Z)], ∂*H*_*max*_/∂E[ln(Z)] = *λ*_*2*_, and ∂*H*_*max*_/∂{*λ*_*2*_E[ln *z*]} = 1. That is, changes in entropy due to changes in process autocorrelation are equal to the product, positive or negative, of *λ*_2_ and *E*[ln *Z*], the two of which covary with process autocorrelation. Therefore, the entropy of the first-order outcome size distribution approaches that of the Weibull distribution, *H*_max_ = *λ*_0_ + *λ*_1_ *E*[*Z* ^*η*^]+(*η*−1)E[ln(*Z*)], continuously with changes in *λ*_*2*_E[ln(*Z*)] and, by inference, autocorrelation of incremental rates, assuming exponentially-distributed incremental rates. In that case, a sequence of outcomes of first-order kinetic processes having incremental rates distributed with entropy constrained only by finite positive mean, converges in distribution from one with tail asymptotically Weibull, to the Weibull, as increment autocorrelation increases from perfect independence to perfect autocorrelation and as the entropies of elemental cause size distributions are maximized.♦

### Demonstration by simulation and meaning of Weibull distribution exponent

To demonstrate convergence of the distribution of first order outcomes to the Weibull distribution as generally as possible for varying autocorrelation, cause magnitudes were sampled as randomly as possible over semi-infinite range, subject to finite positive mean. That is, cause sizes were sampled from independent, partially-correlated, and perfectly correlated (identical) standard exponential distributions, and multiplied. Resulting distributions were tested for GOF versus the two common, semi-infinite, two-parameter distributions having finite moments, i.e. the Weibull and lognormal distributions, and the Pareto I power law distribution.

**Fig 1:**
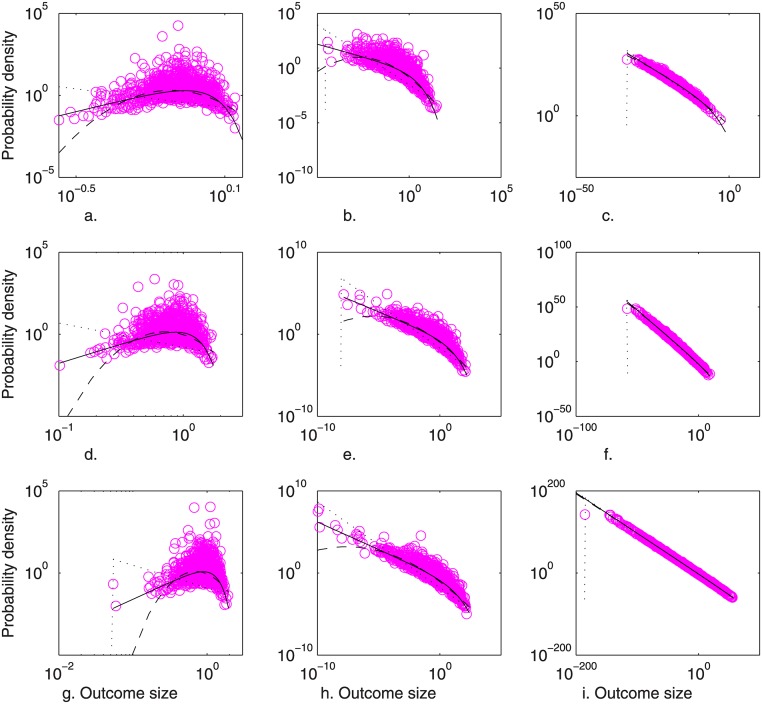
Simulated (o) and fitted Weibull (––), lognormal (—), and Pareto I (^…^) distributions of the products of perfectly independent standard exponential RVs, *c*_i'_'. Panels were simulated as (a)exp∑i=13ln(ci')1/9, (b)∏i=13ci', and (c)∏i=170ci'; the products of partially correlated standard exponential RVs, *c’'*_*i*_, simulated as (d)exp∑i=13ln(ci'')1/9, (e)∏i=13ci'', and (f)∏i=170ci''; and the products of perfectly correlated (identical) standard exponential RVs, *c*_*i*_''', simulated as (g) *c*_*i*_'''^1/3^, (h) *c*_*i*_'''^3^, and (i) *c*_*i*_'''^70^. [Conditions: *N* = 1000, MLE parameter estimates]

In Fig 1, the distributions of products of exponential RVs are shown together with fitted Weibull distributions. The values *η* = 1/3 with *J* = 3, and *η* = 1/70 with *J* = 70 were selected to cover an (unrealistically) broad range in terms of the number of distinct causes (e.g. organs with differing kinetic characteristics), or first-order compounding increments. Further, Weibull distributions often have values of *η* > 1, in which case the distribution is non-monotonic. Therefore, “continuously” compounded first-order processes having *η* = *J* = 3 were also simulated, by assuming a compounding interval equal to 1/9. That is, this case was simulated by a conceptually new method based on Eqs 4 and 5, as $\prod_{j=1}^{3}c_{j}{}^{1/9}$ = $\text{exp}\sum_{i=1}^{3}(1/9)\text{ln}(c_{i})$, in which Δ*t* = 1/9 is the first-order compounding interval and *J* = 3 is the period of continuous compounding (or, equivalently, the number of discrete compounding increments), to simulate values of *η* = 1/ (*J*Δ*t*) > 1. Summarizing specifically, the products of perfectly independent standard exponential RVs, *c*_i'_', were simulated as (a)∏j=13(cj')1/9, (b)∏j=13cj', and (c)∏j=170cj'. Products of partially correlated standard exponential RVs, *c*_*j*_ *''*, were likewise simulated as (d)∏j=13(cj'')1/9, (e)∏j=13cj'', and (f)∏j=170cj''. Products of perfectly correlated (identical) standard exponential RVs, *c*_*j*_''', were simulated as (g) *c*_*j*_'''^1/3^, (h) *c*_*j*_'''^3^, and (i) *c*_*j*_'''^70^.

By inspection, the Weibull distribution fit the simulated first-order outcome sizes well, whereas the lognormal underestimated the probability of small severities. Similar results were obtained when input distributions (a) varied by orders of magnitude in scale, and (b) varied in distribution, i.e. by standard normal distributions left-truncated at zero (data not shown). Fits of the Pareto I, though appearing visually close for *J* = 70, were rejected by the chi-squared test (*α* = 0.05) in 100 of 100 simulations for all cases examined. Results suggest, for example, that the distribution of tumor magnitudes across a population which initiated simultaneously would be Weibull-distributed, given that normally functioning (or wild-type) genes are the most probable (i.e., monotonic elementary cause magnitude distributions) and that rates (probabilities) of expression of multiple mutations of genes involved in cell cycle regulation and apoptosis compound multiplicatively over time.

**Fig 2:**
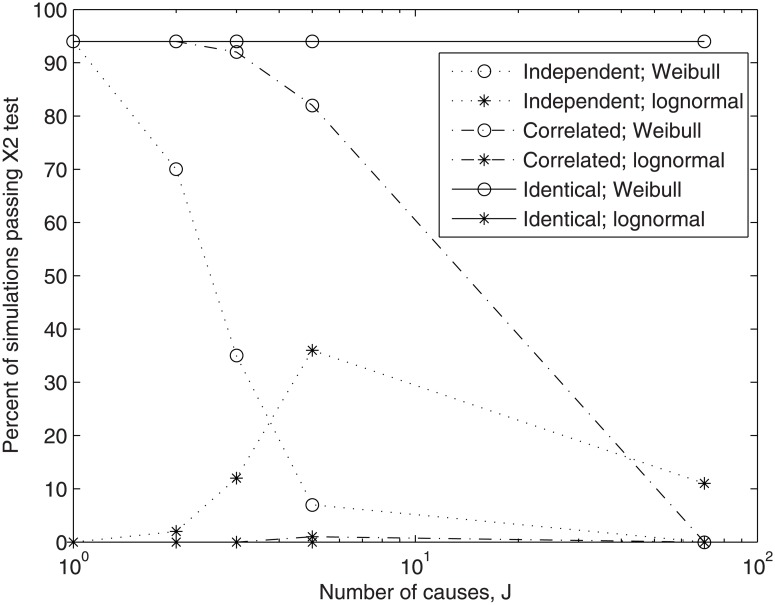
The number of simulated distributions passing a chi squared test for fit to the Weibull and lognormal distributions, as a function of the number of multiplicative asymptotic exponential causes and causal dependence. [Conditions: 1000 sets of *N* = 1000 simulated data points, *α* = 0.05].

In Fig 2, the fits of the Weibull and lognormal distributions are shown as functions of the number of first-order compounding increments, *J*, and increment autocorrelation. As shown, the Weibull distribution was well demonstrated for products of one to five correlated cause sizes. Increased correlation corresponded with improved fit, and fit to larger values of *J*. The lognormal was not demonstrated in general. In light of the dependence of entropy on E[ln(*z*)] shown by Eq 11, typical case (e) was re-run with exponential distributions having means of 100 and 0.01 (representing greater than ±6 logs of scale in *z*). The result was 87 (0) and 90 (1) of 100 passing fit to the Weibull (lognormal), respectively, indicating the Weibull result is robust to the scale of measurement, for typical values of *η*.

Results of Figs 1 and 2 corroborate the derivation of the previous section by illuminating the physical meaning of *η*. That is, fitted parameter *η* decreased to (*J*Δ*t*)^-1^ as cause size correlation increased. For example, *η-*values fitted to one simulation were as follows: (a) 4.6531 >> 9/3, (b) 0.5296 >> 1/3, (c) 0.0976 >> 1/70, (d) 3.3972 > 9/3, (e) 0.3779 > 1/3, (f) 0.0433 > 1/70, (g) 2.9949 ≅ 9/3, (h) 0.3308 ≅ 1/3, and (i) 0.0142 ≅ 1/70. As seen, values of *η* decreased with decreasing causal independence. Furthermore, while Δ*t* strongly influenced the shape of the distribution of outcomes (Fig 1), it did not affect the fit of the Weibull distribution to this shape (Fig 2). For example, cases (a) and (b), both assuming *J* = 3 independent increments, were both accepted by the chi-squared test at the identical rate, though differing drastically in scale due to the difference in Δ*t*, and therefore *η*. Hence, both cases lie on the same lines (independent/Weibull and independent/lognormal) in Fig 2. Similarly, cases (d) and (e) were accepted at identical rates, as were cases (g) and (h). Therefore, generalizing on the result shown in the derivation presented in the previous section, the Weibull exponent parameter reflects the number, *J*, of discrete first-order compounding increments or, equivalently, the continuous compounding period, *T*, along with the width of the interval of first-order compounding, Δ*t*, and is increased to account for imperfect increment autocorrelation. Of course, Weibull parameters *η* and *ξ* also reflect any deviation of the forms of the cause size distributions from the exponential. In any case, overall, *η* may be interpreted as the *log-scale* of outcome-size.

### Emergent property of the Weibull and Fréchet distributions

Based on the foregoing derivation and simulations, the multiplication of non-numerous random cause sizes produces Weibull distributions of outcome size due to autocorrelation and entropic forcing of elementary cause size distributions. This property of distributions of autocorrelated first-order outcomes is termed here *emergence*, defined for discrete process increments as follows:

Let *C*_*j*_^*n*^ = *C*_1_^*n*^, *C*_2_^*n*^, …, *C*_J_^*n*^ be a sequence of *n* series of *J* random variables having respective densities f(**θ**_*j*_)^*n*^, where **θ**_*j*_ = (**θ**_1_, **θ**_2_, …, **θ**_J_) is a parameter vector. Let E[g_1_(*C*_*j*_^*n*^)] = μ_1,*j*_, E[g_2_(*C*_*j*_^*n*^)] = μ _2,*j*_, … E[g_K_(*C*_*j*_^*n*^)] = μ _*K*,*j*_ be constraints on the Shannon entropy of the f(**θ**_*j*_)^*n*^, in which E[.] is the expectation operator, the g_*k*_ = *g*_1_, *g*_2_, …, *g*_*K*_ are continuous functions, the μ _*k*,*j*_ are constants, and 0 < *k* < ∞. Let *C*_*b*_^*n*^, *C*_*b*+1_^*n*^, …, C_*b+B*_^*n*^ be a (*B*+1)-member subset of *C*_1_^*n*^, *C*_2_^*n*^, …, *C*_J_^*n*^. Let **R**_*B*_^*n*^ be the correlation matrix of *C*_1_^*n*^, *C*_2_^*n*^, …, *C*_*B*_^*n*^, **T**_*B*_ be a *B* x *B* matrix having all elements equal to 1, and **R**_*B*_^*n*^ → **T**_*B*_ as *n* → ∞. Let f(**θ**_*j*_)^*n*^ be a special case of f(**θ**_k_)^*n*^, such that one or more values of the parameter vector, **θ**_k_, is equal to h(*B*), in which h is a continuous function, and the corresponding values of the parameter vectors **θ**_*j*_ are equal to h(1). Let the Shannon entropy of the f(**θ**_*j*_)^*n*^ approach a maximum subject to E[g_1_(*C*_*j*_^*n*^)] = μ _1,*j*_, E[g_2_(*C*_*j*_^*n*^)] = μ _2,*j*_, … E[g_K_(*C*_*j*_^*n*^)] = μ _*K*,*j*_, as *n* → ∞. Then, if the product $\prod_{j=b}^{b+B}C_{j}^{\;n}$ converges in distribution to f(**θ**_k_) for all *B* as *n* → ∞, f(**θ**_k_) is emergent.

and analogously for continuous process increments as follows:

Let C_*J*_^*n*^ be a sequence of *n* random integrable functions on R = [0, *J*], 0 < *J* < ∞. Let C_*J*_^*n*^ be distributed everywhere with probability density function f(**θ**_*j*_)^*n*^, where **θ**_*j*_ is a set of functions on R representing the vector of parameters of f(**θ**_*j*_). Let E[g_1_(*C*_*j*_^*n*^)] = μ_1,*j*_, E[g_2_(*C*_*j*_^*n*^)] = μ _2,*j*_, … E[g_K_(*C*_*j*_^*n*^)] = μ _*K*,*j*_ be constraints on the Shannon entropy of the f(**θ**_*j*_)^*n*^, in which E[.] is the expectation operator, the g_*k*_ = *g*_1_, *g*_2_, …, *g*_*K*_ are continuous functions, the μ _*k*,*j*_ are constants, and 0 < *k* < ∞. Let C_*B*_^*n*^ be the segment of C_*J*_^*n*^ on [*b*, *B*], 0 ≤ *b* < B ≤ *J*. Let **R**_*B*_^*n*^ be the correlation functions of the C_*B*_^*n*^, and **R**_*B*_^*n*^ → 1 as n → ∞. Let f(**θ**_*j*_)^*n*^ be a special case of f(**θ**_k_)^*n*^, such that one or more values of the parameter vector, **θ**_k_, is equal to h(*B*), in which h is a continuous function, and the corresponding values of **θ**_*j*_ are equal to h(1). Let the Shannon entropy of the f(**θ**_*j*_)^*n*^ approach a maximum subject to E[g_1_(*C*_*j*_^*n*^)] = μ _1,*j*_, E[g_2_(*C*_*j*_^*n*^)] = μ _2,*j*_, … E[g_K_(*C*_*j*_^*n*^)] = μ _*K*,*j*_, everywhere for all lags, *τ*, as n → ∞. Then, if the product integral $\pi_{b}^{B}C_{B}{}^{dB}$ converges in distribution to f(**θ**_k_) for all C_*B*_, f(**θ**_k_) is emergent.

Informally, when a discrete or continuous series of correlated RVs, or any subset thereof, all coming from a common, asymptotically-maximum entropy distribution (by implication, arising in nature as a result of the tendency towards higher entropy) are multiplied (e.g., in a physical process), the resulting outcome size converges in distribution to a consistent, or emergent, form as (natural) autocorrelation of the series increases, and as the entropy of the distributions of the correlated RVs increases subject to prevailing (physical) constraint(s). Emergence implies that products of autocorrelated, asymptotically exponential RVs are distributed asymptotically Weibull, and that products of such Weibull products are also asymptotically Weibull-distributed. Also, because reciprocals of Weibull RVs are distributed Fréchet [36], the Fréchet distribution can emerge via first-order processes. Other distributions, such as the lognormal and Pareto I, for which powers of the RV have the parent distribution by transformation of variables, are possible candidates for mathematical emergence. However, the required constraints on the entropy of their elementary cause size distributions may not represent typical physical systems closely.

### Demonstration versus simulated severity distributions

**Fig 3:**
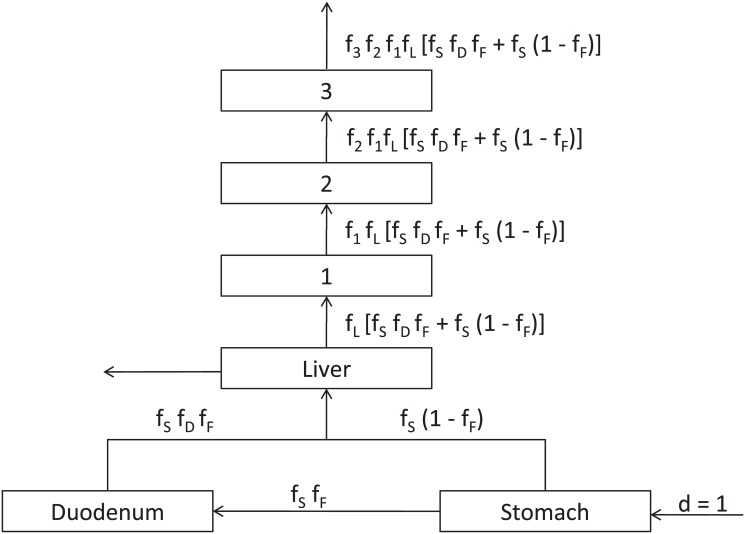
Definition diagram for a generalized, physiologically-based, first-order model of a liver-mediated toxicological pathway, including a terminal series of three generalized pharmacodynamic steps.

Because elimination and biotransformation rates vary across a population and in time, a distribution of chronic toxicant concentrations, each an autocorrelated first-order (Weibull) outcome, may be realized at the target tissue. For example, Fig 3 depicts the mass fraction of a dose of hydrophilic toxicant passing the stomach to the duodenum and liver and, following partial elimination (primarily from the liver), on to three pharmacodynamic reactions terminating in illness. (Of course stomach to duodenum transfer is direct, whereas e.g. the duodenum-to-liver pathway may include diffusion, toxin-protein binding, active energy-dependent transfer, a function of intestinal mucosal blood flow, and other mechanisms.) Final normalized illness severity at a fixed dose, *A*_*0*_, can be expressed in a first-order PBPD sense as:

$$A/A_{0}=f_{3}f_{2}f_{1}f_{L}[f_{S}f_{D}f_{F}+f_{S}(1-f_{F})],$$ (12)

in which *f*_*S*_ is the fraction passed by the stomach, *f*_*F*_ is the rate of distribution of *f*_*S*_ to the duodenum, *f*_*D*_ is the further fraction passed to the liver, *f*_*L*_ is the fraction passed by the liver, and *f*_*1*_, *f*_*2*_, and *f*_*3*_ are generalized intermediate metabolic fractions representing pharmacodynamic interactions. Alternatively, the model could be simplified to account for principal steps only. For example, if the fraction *f*_*F*_ is either large or small, a basic model might be:

$$A/A_{0}=f_{1}f_{L}$$ (13)

**Fig 4:**
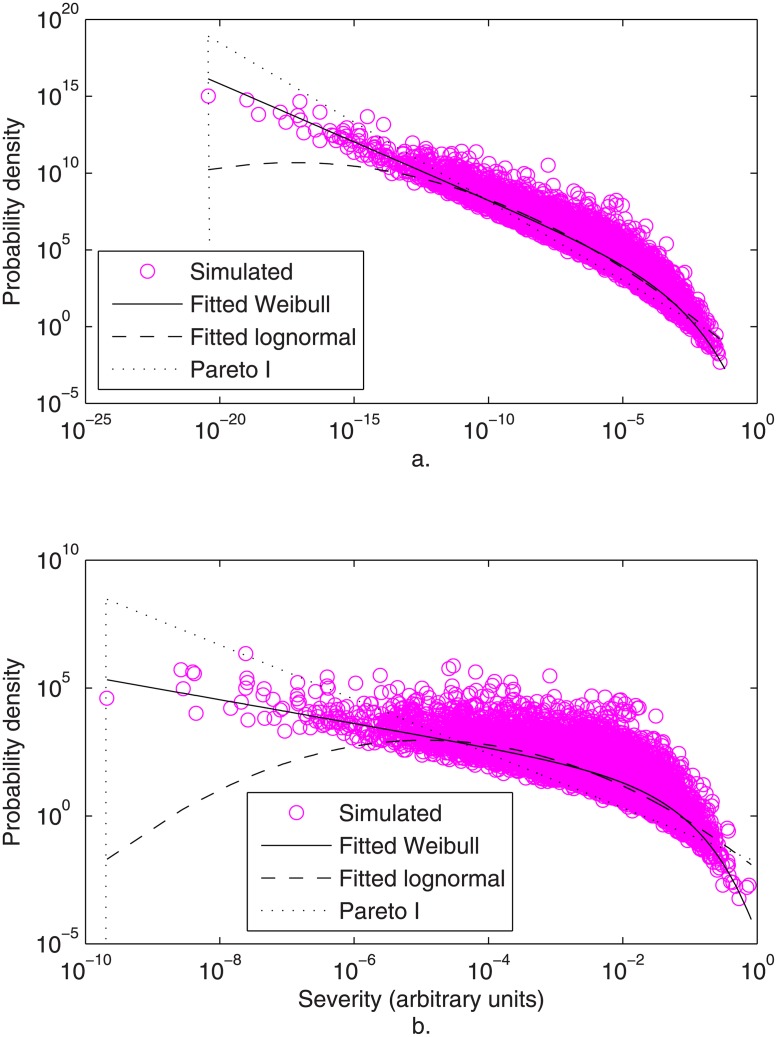
Simulated (o) and fitted Weibull (––) and lognormal (—) distributions of illness severity. Severities were simulated as (a) the results of the multiplicative model of illness severities of Eq 12, and (b) the results of the simplified multiplicative model of illness severities of Eq 13. [Conditions: all fractions, *f*_*X*_, distributed standard exponential, *N* = 100,000; subsequent fractions correlated by copula; MLE parameter fits]

The results of Eqs 12 and 13 are plotted in Fig 4(a) and 4(b), respectively, assuming fractions passed, *f*_*X*_, to be distributed exponential with mean 0.1. GOF results are shown in Table 1. The Weibull distribution was accepted consistently, in contrast with the lognormal and Pareto I. Such multiplicative models could apply as well to e.g. probabilities of mutations that would collectively result in uncontrolled cell proliferation or malignant transformation.

**Table 1:** Simulation GOF results.

Simulation	Distribution	GOF*
PB-PD model, Eq 12, *f_X_*, exponential with mean 0.1	Weibull	93/100
	Lognormal	0/100
	Pareto I	0/100
Simplified first-order PB-PD model, Eq 13, *f_X_*, exponential with mean 0.1	Weibull	94/100
	Lognormal	0/100
	Pareto I	0/100
Metabolic network model, constant toxicant dose, Fig 5(a)	Weibull	*p* = 0.31
Metabolic network model, variable toxicant dose, Fig 5(b)	Shifted Weibull	*p* = 0.80
Michaelis-Menten saturation kinetic outcomes, Eq 14, *z_0_* = 50, *K* = 25, E[*r'*] = -7.5, *t* = 1~10, *r'* ~ correlated standard exponential	Weibull	97/100
	Lognormal	0/100
	Pareto I	visually log-log non-linear
Michaelis-Menten saturation kinetic outcomes, Eq 14, *z_0_* = 50, *K* = 25, E[*r'*] = -7.5, *t* = 1~10, *r'* ~ identical standard exponential	Weibull	30/100
	Lognormal	0/100
	Pareto I	visually log-log non-linear

*χ^2^ test results,

α > 0.05: number of simulations passing/total simulations, or *p*-value.

### Demonstration versus simulated metabolic network models

Following the arrival of a Weibull distribution of concentration at the target tissue, smaller-scale pharmacodynamic interactions may occur by parallel and network pathways, which may collectively result in an aggregate process that proceeds generally in proportion to prevailing concentration, i.e. by overall autocorrelated first-order kinetics. In fact, metabolic, protein, and regulatory networks have been shown to have largely “scale-free,” or power law-like degree distributions (of the number of connections per vertex) [10,37], as a result of largely (but not wholly) preferential attachment of new network nodes to the more highly connected existing nodes [38]. Moreover, the preferential attachment model can be viewed as one of autocorrelated first-order kinetic growth, as shown in S1 File.

Basic simulations of severity distributions resulting from chemical attack on a biological network were conducted as follows. First, undirected networks constructed with 80% preferential/20% uniform attachment of new vertices, starting with 10 initial vertices, each having 0.1 probability of connection initially, were assumed as generalized test models of the target network. Because the architecture of individual biological networks has been found highly dynamic and variable [39], average vertex degree among the simulated networks (trials) was assumed to vary according to a normal (*μ* = 25, *σ* = 5) distribution (average degree must be defined for each simulation to define a stopping point for new connections). Then the size, *S*, of the largest cluster of connected vertices following attack on the network hubs by a system-specific toxicant was studied, as a measure of the remaining integrity in a network. That is, the difference in *S* was measured in fifty 1000-vertex networks after removal of five of the 10 initial (generally highest-degree) network vertices, simulating strong chemical toxicity targeting hubs of the network controlling the physiological function affected. Because each hub removed tends to be successively smaller in degree, due to removal of links to previously removed hubs, the process is roughly first-order.

**Fig 5:**
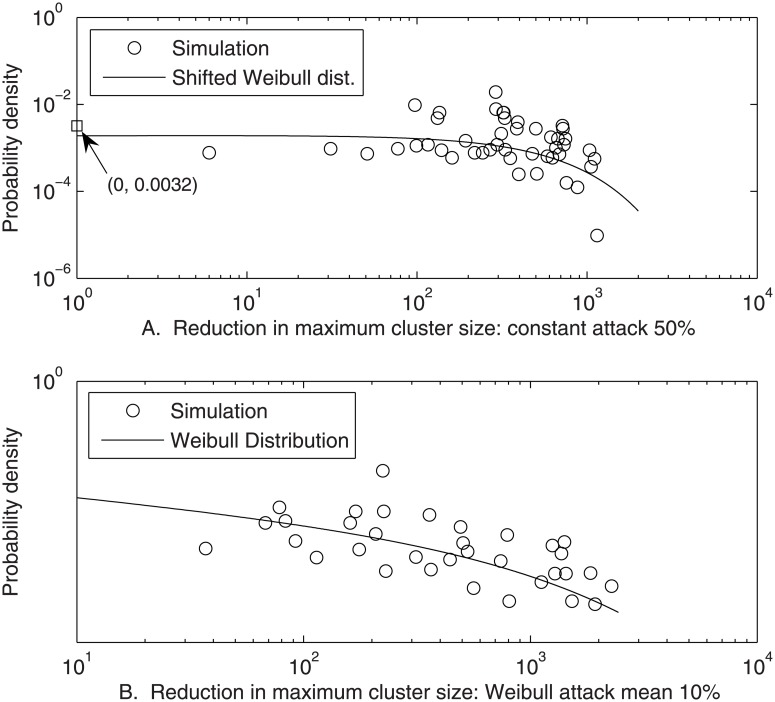
Simulated illness severities, Δ*S*, defined as reduction in the size of the largest cluster remaining after removal of some of the most connected vertices of 50 1000-vertex scale-free networks, and fitted Weibull distributions. Severities were simulated as (a) Δ*S*—162 after removal of 5 vertices, and (b) Δ*S* after removal of discrete Weibull-distributed numbers of vertices averaging 5. [Conditions: average network connectivity distributed normal (25, 5); MLE parameter fits]

In Fig 5(a) and Table 1, the distribution of resulting severities, Δ*S*, is shown. No significant difference from the shifted, three-parameter Weibull distribution with threshold, 162, equal to the minimum Δ*S* (*N* = 50; *α* = 0.05; *p* = 0.31) was found. Note that distributions obtained were characteristically noisy, like real biological data, and difficult to fit with 95% confidence to any distribution. However, the same networks were then restored and subjected to removal of discrete Weibull (mean 5, parameters *ξ* = 0.70, *η* = 0.74)-distributed [17] numbers of the 10+ initial vertices. This variability modeled a variable concentration of toxicant reaching the target organ. The distribution of resulting severities, shown in Fig 5(b) and Table 1, was fitted by the Weibull distribution (*N* = 50; *α* = 0.05; *p* = 0.80). Biological networks are not yet well-understood, and the assumptions of the simulations presented, particularly the percent preferential attachment of new nodes, affect the log-log linearity of the resulting distribution. Therefore, definitive comparison of the Weibull distribution with competitive distributions was not attempted. However, results suggest Weibull-like distributions of severity may result from chemical attack on such metabolic networks.

### Demonstration versus simulated outcomes of saturation kinetics

Acute toxicants (and pharmaceuticals) may not be the limiting reactant in physiological systems, in which case pseudo-first-order kinetics may not apply. Rather, acute toxicants may act according to saturation kinetics, generally the Michaelis-Menten (Monod) rate law, in which the rate of change in concentration is asymptotically first-order at low concentration, increasing to a maximum, zero order rate at high concentration. Similarly, generalized saturation kinetics may apply to the rate of change in illness severity. The Michaelis-Menten integrates as follows:

$$\begin{array}{l} \frac{dz}{dt}=\frac{r'z}{K+z}\\ {\left(\frac{z}{z_{0}}\right)}^{K}e^{z-z_{0}}=e^{r't} \end{array}$$ (14)

In Eq 14, *K* is the positive real half-saturation constant, *r'* is the real maximum-rate constant, *Z*_*0*_ is the initial magnitude, e^*r'*^ is the exponentially-distributed growth/decay multiplier. Then, the distribution of saturation-kinetic outcomes, when e^*r't*^ is distributed Weibull (*ξ*, *η*) and *K* is constant, can be found by transformation of variables as:

$$\begin{array}{l} \text{F(z)}=\text{1-exp}\left[-{\left(\frac{z^{K}e^{z}}{\xi }\right)}^{\eta }\right]\;,\;0<z<\infty \\ \text{f(}z\text{)}=\eta \left(\frac{K}{z}+1\right){\left(\frac{z^{K}e^{z}}{\xi }\right)}^{\eta }\text{exp}\left[-{\left(\frac{z^{K}e^{z}}{\xi }\right)}^{\eta }\right] \end{array}$$ (15)

Importantly, a deterministic saturation process may be approximated by an autocorrelated first-order process for which the rate constant decreases as reactant increases. In fact, the relationship is seen to be exact if the first-order rate constant decreases smoothly with concentration as *r* = *r'*/(*K* + *C*). This curvilinear relationship, representing perfect dependence though not perfect autocorrelation, suggests that the Weibull distribution may adequately model common saturation kinetic rate laws. Moreover, in toxicological applications, particularly to chronic toxicants, little or no saturation is expected. Therefore, while the three-parameter univariate distribution of Eq 15 may be useful in pharmaceutical applications, the two-parameter Weibull may be applicable even after the onset of saturation, potentially valuable to minimize data requirements in many applications.

**Fig 6:**
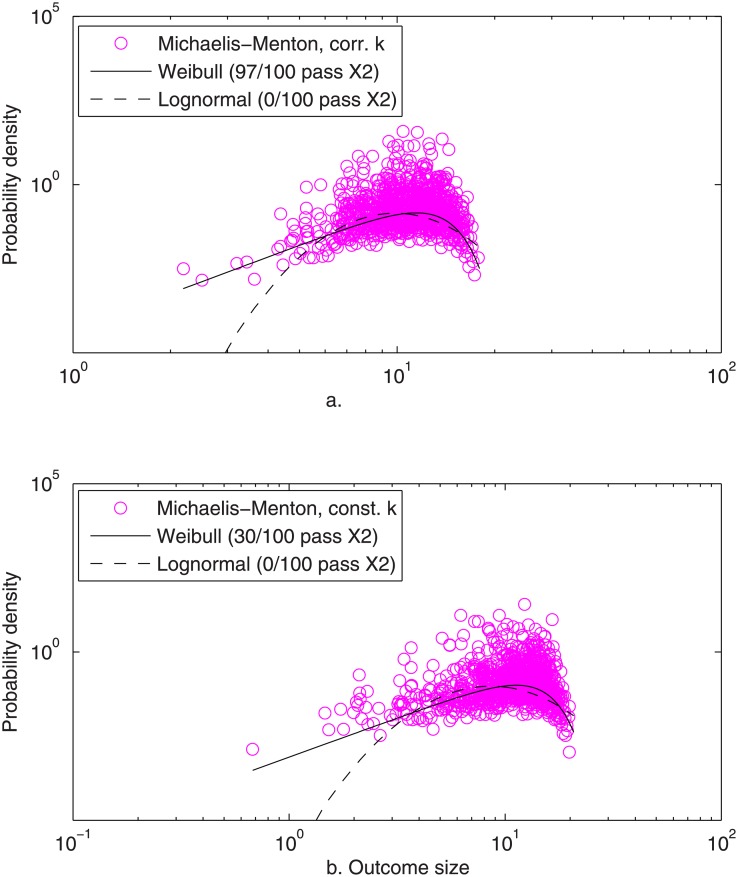
Simulated (o) and fitted Weibull (––) and lognormal (—) distributions of the outcomes of Michaelis-Menten saturation kinetics, with maximum-rate constant simulated as (a) partially correlated standard exponential RVs, and (b) perfectly correlated (identical) standard exponential RVs, both simulated as described for Fig 1. [Conditions: *N* = 1000, MLE parameter estimates]

To examine the empirical applicability of the Weibull and lognormal distributions to the outcomes of Michaelis-Menten kinetics when e^*r'*^ varies across a population, fit to the Michaelis-Menten rate law was tested, for *z*_*0*_ = 50, *K* = 25, E[*r'*] = -7.5, and *t* = 1~10. This case was selected such that the first five kinetic steps are more nearly zero order, and the final five are more nearly first-order. Simulation was by the differential form of Eq 14, and random *r'* was generated as described for Fig 1. That is, d*z*_*j*_ was computed as *r'z*_*i*_/(*K* + *z*_*i*_) in each *i*-th of 10 time steps, and used to computed *z*_*i+1*_ = *z*_*i*_ + d*z*_*i*_. As shown in Fig 6 and Table 1, the Weibull distribution fit the case of correlated *r'* in 97 of 100 simulations (*χ* ^*2*^ *α* = 0.05), whereas the lognormal and Pareto I fit in none. Even in the extreme case of perfect (linear) correlation among time increments, i.e. *r'* constant with respect to time, the Weibull distribution fit 30 of 100 simulated outcome sizes, whereas the lognormal again fit none. Therefore, the Weibull form appears initially to be robust to the onset of saturated kinetics.

### Demonstration versus available empirical data: noncarcinogen

The generality of the Weibull form has been well-demonstrated versus empirical data on a broad range of natural and anthropogenic complex system outcomes previously, and the distributions of Fig 1 may cover the range of shapes of many such reported heavy-tailed distributions. However, the Weibull distribution has not been studied with respect to illness severities.

**Fig 7:**
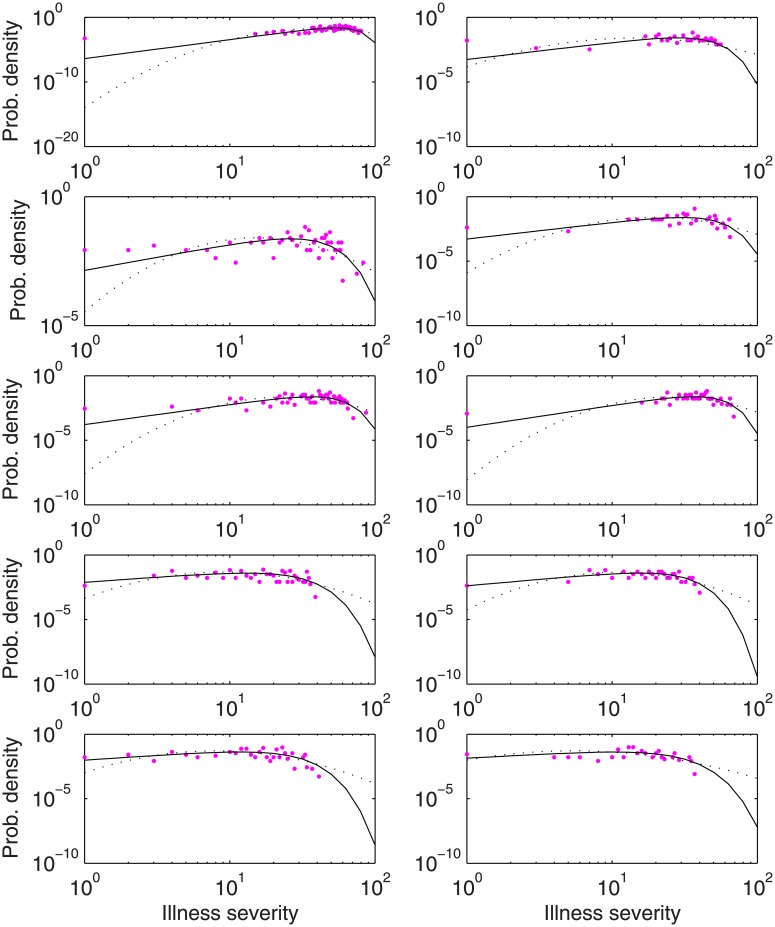
Empirical distributions (o) of test score differences (maximum group score minus individual score plus one) in children born of mothers of varying hair mercury during pregnancy, and fitted Weibull (−) and lognormal (—) distributions. Rows 1–5: TOLD_SL, WISC_RP, WISC_RF, MCC_PP and MCC_MOT, respectively; columns 1–2: 6–87 and 3–5.99 mg/kg hair mercury, respectively.

To demonstrate the Weibull distribution empirically for illness severity, data of Kjellström and co-workers on children of mothers having varying concentrations of hair mercury during pregnancy [40,41] were obtained. To examine possible neurotoxicity of mercury on the children, results of five scholastic and psychological tests, abbreviated as TOLD_SL, WISC_RP, WISC_RF, MCC_PP and MCC_MOT (data shown in S1 Table), were related to maternal hair mercury. Four matched dose groups were identified by the authors: 6–87, 3–5.99, 0.1–2.99, and 0.1–2.99 mg/kg hair mercury. Groups were used by the authors to develop a relationship between mercury dose and population health response, and were used here to evaluate the form of the distributions of illness severity at each approximately-constant dose. The last two, considered control groups by the authors, are not considered here. Illness severity is defined here as the difference between the maximum score of a group and each individual score in that group, plus unity. The fits of the illness severity distributions to the Weibull and lognormal forms, for the five test scores and two dose groups, are shown in Table 2 and Fig 7.

**Table 2:** Fits of the Data of Kjellström et al. on Test Scores of Children with Prenatal Mercury Exposure to the Weibull and Lognormal Distributions

		6–87 mg/kg hair Hg*		3–5.99 mg/kg hair Hg*	
Test	*p*	Weibull	Lognormal	Weibull	Lognormal
TOLD_SL	X2	0.2647	1.0597e-7	0.6491	1.4248e-6
	KS	0.4084	0.0011	0.4373	0.0054
	*η*	3.9328	-	2.3261	-
WISC_RP	X2	0.0122	6.2639e-6	5.2516e-4	0.0011
	KS	0.2115	0.0013	0.2454	0.0269
	*η*	2.0188	-	2.2945	-
WISC_RF	X2	0.8913	0.0059	0.4211	3.5344e-4
	KS	0.8196	0.0796	0.6584	0.0570
	*η*	2.5488	-	2.6893	-
MCC_PP	X2	0.4857	6.2089e-5	0.4199	0.0306
	KS	0.6737	0.0757	0.9727	0.5275
	*η*	1.8027	-	2.0073	-
MCC_MOT	X2	0.2525	0.0210	0.1574	0.0028
	KS	0.9103	0.2486	0.1166	0.0016
	*η*	1.7677	-	1.6261	-

*The Pareto I distribution was not fitted due to visual log-log nonlinearity of all plots

The severity distributions are visually similar to the simulated distribution of Fig 1(a), and again the Weibull distribution fits were superior, particularly at low severities where the lognormal is approaching zero probability. By the KS test, no Weibull distribution fits were rejected, whereas 10 of 15 lognormal fits were rejected. By the more rigorous chi squared test, the lognormal was rejected in all cases, whereas the Weibull distribution was accepted (*p* = 0.05) in 8 of 10 cases. Of note, the Weibull distribution was also strongly favored in plots of the combined control groups, as well (data not shown). The Pareto I distribution was not fitted due to the obvious visual log-log nonlinearity of all plots.

### Demonstration versus available empirical data: carcinogen

Dose response data on carcinogens do not generally reflect a numeric severity, except in terms of time-to-tumor. That is, tumor weights, for example, are not generally recorded. Statistical analysis of time-to-tumor data was reviewed by Krewski and Brown [42]. Perhaps most widely applied has been the Multistage Weibull model [42], a multivariate model in which the dose-response function is assumed to be an exponential distribution on a product of a power of time-to-tumor and a polynomial of the dose of a toxicant. At a constant dose, this model implies a Weibull distribution of time-to-tumor. However, more recently tumor growth kinetics have been proposed to be first-order, using rescaled dimensionless mass and time variables based on principles of allometric scaling [43,44].

Assuming first-order tumor growth kinetics, time-to-tumor is related to tumor mass/severity by z* = (e^*r*^ *)* ^*τ*^, in which z* is a constant unitless tumor size (e.g. mass) corresponding to positive diagnosis (e.g. neoplasm or carcinoma), *τ* is the time-to-positive-diagnosis, and *r* is the growth rate constant. In that case, if (e^*r*^) is an exponentially-distributed cause size, then the *time-transform* distribution of *τ* can be found, by transformation of variables and recognition of the result as the distribution of the reciprocal of a Gompertz random variate, as:

$$\begin{array}{l} \text{f}(\tau )=\frac{\phi \theta \text{exp}(\phi /t)}{\tau^{2}\text{exp}\left\{\theta \left[\text{exp}(\phi /\tau )-1\right]\right\}}\\ \text{F}(\tau )=\text{exp}\left\{-\theta \left[\text{exp}(\phi /t)-1\right]\right\} \end{array}$$ (16)

Assuming saturation kinetics proceeding to a fixed observable tumor size, z*, the relationship between *e* ^*-r'*^ and *t* is the same as for the first-order case, as follows:

$${\left(\frac{z*}{z_{0}}\right)}^{K}e^{z*-z_{0}}={\left(e^{r'}\right)}^{\tau }$$ (17)

Hence, by the autocorrelated first-order model, time-to-tumor is distributed by Eq 14 whether first-order or saturation kinetics apply.

The ED_01_ project, in which >24,000 female BALB/c StCr/fC3Hf/Nctr mice were exposed to the carcinogen 2-acetylaminofluoren [45], has provided perhaps the most complete dataset for analysis of time-to-tumor models. In the constituent studies of the ED_01_ project found useful for assessing the applicability of Eq 16, feed concentration was held constant at selected doses, dosing was continuous, and mice were sacrificed and necropsied for bladder neoplasm, liver neoplasms, and bladder carcinomas at nine selected time intervals. Intervals were chosen such that moribund and dead mice could be similarly necropsied, and counts included with the sacrificed animals at the times nearest their death or removal. In this way, the proportion of all animals having *z* > z* could be computed. Therefore, these data (reproduced in S2 Table) were fitted to the Weibull and time-transform distributions. Data for all dose levels were plotted as such, and based on these plots it was considered that only doses of 75, 100, and 150 ppm produced sufficient positive counts to reasonably assess the fit of the distributions.

**Fig 8:**
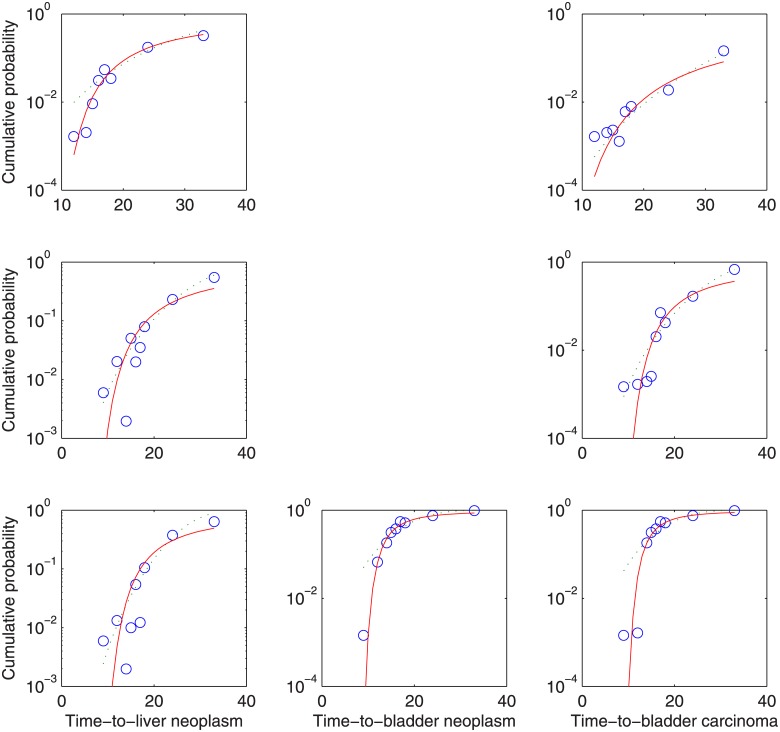
Empirical distributions (o) of ED_01_ study data on bladder neoplasms, liver neoplasms, and bladder carcinomas resulting from continuous exposure to the carcinogen 2-acetylaminofluoren, and fitted Weibull (—) and first-order time-transform (−) distributions. Columns 1–3: liver neoplasms, bladder neoplasms, and bladder carcinomas, respectively; Rows 1–3: 75, 100, and 150 ppm, respectively. Bladder neoplasm and bladder carcinoma data are identical at 75 and 100 ppm (all neoplasms malignant), therefore not shown again.

Fits of the datasets are presented in Table 3 and Fig 8. As shown, the Weibull and time-transform distributions both passed (*p* = 0.05) in only three of seven cases. However, the fit of the time-transform distribution was visually quite good with the exception of the 33-month data points. This increased neoplasia and carcinoma at 33 months relative to those predicted by first-order kinetics may be explained by the advanced age of these mice, which were ca. 65% past their life expectancy [46], and the resulting compromised immune response. Thus the time-transform distribution of time-to-tumor, corresponding to a Weibull distribution of illness severity, was demonstrated within reason, given transformed biological data collected for a different purpose.

**Table 3:** Fits of the ED_01_ Data on Sacrificed, Moribund, and Dead Mice to the Weibull and Time-Transform Distributions.

Endpoint	Dose (ppm in feed)	*p*, Weibull	*p*, time-transform
Bladder neoplasms	75	(same as bladder carcinomas)	
Bladder neoplasms	100	(same as bladder carcinomas)	
Bladder neoplasms	150	9.2972e-008	0.2469
Liver neoplasms	75	0.0055	0.3494
Liver neoplasms	100	0.1502	7.7304e-004
Liver neoplasms	150	0.0021	3.8176e-008
Bladder carcinomas	75	0.8366	0.2631
Bladder carcinomas	100	0.3100	7.9544e-004
Bladder carcinomas	150	3.3529e-014	0.0060

## Discussion and Conclusions

As can be seen in the simulations presented, the Weibull distribution can plot essentially log-log linear over wide range, suggesting that it may sometimes be mistaken for a power law. In fact, linearity is increased further when *η* varies, either within systems or in datasets pooled across systems, as shown in S2 File. Of note, the generality of first order mechanisms may also explain the fact that similar, apparently log-log linear distributions are generally obtained regardless of the units of analysis of outcome size (e.g., mass of gas released explosively, dollars of damage, or mortality) [47], and are further generally obtained for all subsets of the data analyzed, (e.g. refinery explosions, explosions occurring from 2003 through 2012, or explosions in the mid-western US) [48].

The emergence of the Weibull and Fréchet distributions is related to multiplication-stability [13], differing in that convergence increases with the autocorrelation and entropy (e.g., exponential character) of elements rather than with their independence and multiplicity. The simulation results of Fig 2 suggest that as the number of first-order compounding increments becomes large, the form of the outcome size deviates from the Weibull, unless autocorrelation among increments is perfect. However, in some physical systems an emergent distribution may be an attracting form, increasing in convergence with increasing increment numbers, because as outcome magnitude evolves multiplicatively toward more extreme values, these values may become either much larger or much smaller than subsequent control mechanisms can contain, leading to increased correlation among cause magnitudes that increasingly represent failures of essentially naught or 100%. For example, organs may easily assimilate a low-level stressor, or fail completely at a high level.

At first the term emergence may seem overly general for a property specific to first-order processes. However, first-order systems are apparently quite general in complex systems; consider that at least the two well-known mechanistic models of complex systems which produce outcome sizes having scaling distributions, self-organized criticality [49] and preferential growth [10], can be viewed as particular cases of autocorrelated first-order kinetics, as explained in S1 File. Also, the generality of the result may be further enhanced in predominantly first-order systems because sums of perfectly correlated RVs of the same distribution, up to a change in scale, have the parent distribution. That is, we may expect that sums of correlated Weibull-distributed products, perhaps representing parallel first-order pathways, also tend towards the Weibull distribution.

Taken together, results suggest that the occurrence of the Weibull distribution in autocorrelated multiplicative systems parallels the occurrence of the Gaussian distribution in independent linear (additive) systems. In fact, Johnson et al. [50] note:

“The close agreement that Weibull demonstrated between his observed data and those predicted with the fitted models was quite impressive. … The Weibull distribution is undeniably the distribution that has received maximum attention during the past 23 years [since 1970] … This is clearly evident from the large number of references (most of which have been published since 1970) at the end of this chapter [518 cited].”

They observed further that, in spite of its empirical ubiquity, the only physical meaning attributed to the Weibull distribution had been that of a distribution of failure times in reliability analysis, when the failure (hazard) rate takes certain forms [51]. Subsequently, LaHerrere and Sornette [14] suggested, based on empirical analysis and examination of independent multiplicative systems, that:

“Multiplicative processes often constitute zeroth-order descriptions of a large variety of physical systems, …” and “We do not claim that all power law distributions have to be replaced but that observable curvatures in log-log plots that are often present may signal that another statistical representation, such as a [Weibull distribution], is better suited.”

Based on the derivation and demonstration presented in this paper, the following conclusions can be drawn:

1. The Weibull distribution is derived as the asymptotic distribution of first-order growth/decay kinetic processes. Convergence to the Weibull distribution results from the existence of finite positive mean elementary cause magnitudes, and increases with autocorrelation among increments of a first-order kinetic process. Hence, the distribution is said to be emergent;
2. The generality, dominance, and initial basis of the Weibull distribution shown previously for many natural and anthropogenic complex system outcomes may be explained by the ubiquity of autocorrelated generalized first-order processes in physical systems and resulting Weibull emergence, a property which may engender multiplication stability in some systems;
3. In autocorrelated first-order systems the Weibull distribution, which may appear log-log linear in shape, is suggested to dominate the Pareto I power law distribution. Also in such systems, the emergent result is suggested to dominate multiplicity- and independence-driven convergence of products of identically-distributed RVs to the lognormal distribution (by the Central Limit Theorem);
4. The Weibull exponent parameter represents the number and rates of the first-order compounding increments, the autocorrelation of compounding increments, and the degree to which increments are exponential in distribution. Overall, the lumped exponential parameter (like power law exponents) may be viewed as a logarithmic-scale parameter; and
5. The Weibull distribution represents a general distribution of illness severity, and is shown to be robust to the onset of saturation kinetics in autocorrelated first-order systems.

## Supporting Information

S1 File Self-Organized Criticality and Preferential Attachment as Correlated First-Order Models. (DOCX)

S2 File Log-log Linearity of Weibull Distributions with Variable *η*. (DOCX)

S1 Table Data of Kjellström et al. [40,41], Sorted by Hair Mercury Level, on Childrens’ Test Scores Found Sensitive to Prenatal Mercury Exposure. (DOCX)

S2 Table Time-to-Tumor Data of the ED_01_ Study: Bladder Neoplasms, Liver Neoplasms, and Bladder Carcinomas [45]. (DOCX)
