# Correction: Distributions of Autocorrelated First-Order Kinetic Outcomes: Illness Severity

**DOI:** 10.1371/journal.pone.0174526

**Published:** 2017-03-21

**Authors:** James D. Englehardt

The following paragraph in the “Derivation of the asymptotic distribution to first-order outcomes” section of the results should be omitted: The third constraint of Eq 9 can be seen related to the correlation among the Cj. That is, if the Cj are correlated, then when the Cj tend higher than unity, their product will tend higher still as they become disproportionally more extreme, such that ln[Z] will tend high, and vice versa when the Cj tend low. When the Cj are not correlated, the effect will be reduced, and E[ln(Z)] will tend closer to zero. Thus, the absolute value of E[ln(Z)] tends toward a direct relationship with correlation.

The author offers the following explanation for the removal of the above paragraph: While correlation among cause magnitudes affects individual outcome magnitudes, it does not change the mean log outcome magnitude for a particular first-order process having the fixed mean cause magnitudes, *θ*_*1*_, *θ*_*2*_, …, *θ*_*J*_. This can be seen by expanding the third version of Equation 8 shown on page 10, as follows:

$$E[\text{ln}Z]=E\left[\sum_{j=1}^{J}\text{ln}C_{j}\right]=\sum_{j=1}^{J}E[\text{ln}C_{j}]$$

and noting that the mean log magnitude, E[ln *C*_*j*_], of each cause is fixed by the value of the parameter, *θ*_*j*_, of the distribution of its magnitude, irrespective of cause magnitude correlation. Therefore, the expression E[ln(*Z*)] should be replaced with the expression ln(*Z*) in the last two sentences of the paragraph. Then it should be noted that, for a particular first-order process, E[ln(*Z*)] is a constant with respect to process autocorrelation, having a value determined solely by the mean cause magnitudes, *θ*_*1*_, *θ*_*2*_, …, *θ*_*J*_.

Three sentences in the twelfth paragraph of the “Derivation of the asymptotic distribution of first-order outcomes” section of the Results are incorrect. The correct sentences are: Thus, as process autocorrelation varies, ∂Hmax/∂{λ2E[ln z]} = 1. That is, changes in the entropy of the maximum entropy distribution for a first order process due to changes in process autocorrelation are equal to the product, positive or negative, of λ2 and E[ln Z]. However, from Equation 8, $E[\text{ln }Z]\;=\;\bar{J\text{ ln }G(\theta_{j})}$ is constant with respect to process autocorrelation for a particular first-order process. Therefore, the entropy of the corresponding maximum entropy, and therefore the actual, first-order outcome size distribution varies continuously with *λ*_2_ and therefore with autocorrelation of incremental rates, approaching that of the Weibull distribution, Hmax = λ0 + λ1E[Zη]+(η−1)E[ln(Z)], assuming exponentially-distributed incremental rates.

There are errors in the image and caption for Fig 2. Please see the complete, correct Fig 2 here.

**Fig 2:**
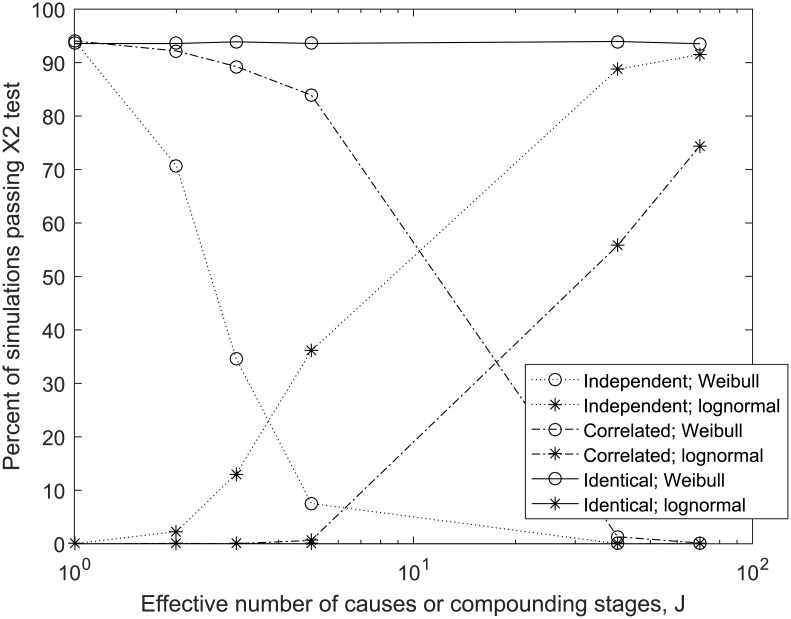
The number of simulated distributions passing a chi squared test for fit to the Weibull and lognormal distributions, as a function of the number of equivalent compounding stages, or multiplicative asymptotic exponential causes, *J*, and causal dependence. [Conditions: 10,000 sets of N = 1000 simulated data points, α = 0.05, Δt = 1].

The fourth paragraph of the “Demonstration by simulation and meaning of Weibull distribution exponent” section of the Results is incorrect. The correct paragraph is: In Fig 2, the fits of the Weibull and lognormal distributions are shown as functions of the number of equivalent first-order compounding increments, *J*, and increment autocorrelation. As shown, the Weibull distribution was well demonstrated for products of up to five correlated cause sizes, i.e. five equivalent correlated stages of compounding, and demonstrated preferable to the lognormal distribution for up to 15–20 correlated causes all having equivalent mean size. Increased correlation corresponded with improved fit, and fit to larger values of *J*. The lognormal was demonstrated only for outcomes having more than ~50 stages of compounding. Confirming the lack of dependence of the form of f(*z*) on E[ln(*z*)] shown by Eq 8, typical case (e) was re-run with exponential distributions having means of 100 and 0.01 (representing greater than ±6 logs of scale in *z*). The result was 87 (0) and 90 (1) of 100 passing fit to the Weibull (lognormal), respectively, indicating the Weibull result is robust to scale, for typical values of *η*.

Point 2 in the Discussion and Conclusions is incorrect. The correct text is: 2. The generality, dominance, and initial basis of the Weibull distribution shown previously for many natural and anthropogenic complex system outcomes may be explained by the ubiquity of autocorrelated generalized first-order processes across scales in physical systems and resulting Weibull emergence, a property which may engender multiplication stability in some systems;
