# Supplementary material for: Distributions of Autocorrelated First-Order Kinetic Outcomes: Illness Severity
Source: PLoS One. 2015 Jun 10;10(6):e0129042. doi: 10.1371/journal.pone.0129042 (PMC4465627; doi:10.1371/journal.pone.0129042)
Supplement: S1 File — (DOCX) [file pone.0129042.s001.docx]

**Self-Organized Criticality and Preferential Attachment as Correlated First-Order Models**

Two well-known and general complex system models producing asymptotically log-log linear distributions of outcome size can be written as autocorrelated first order growth. First, in the self-organized critical (SOC) model of species extinction [1], the least fit species in a 30-species ring, and its two neighbors, are revised with a new uniform random fitness value, from 0 – 1, in each time step. By revising the least fit species in each step, fitness values evolve until all species are above a self-organized critical value, ending the cascade. The model has also been used as one of human pathogenesis, with each species replaced by a physiological process or organ, and the ecosystem replaced by a human subject evolving to health at the end of each illness cascade [2].

SOC cascade lengths can be viewed as an autocorrelated first order outcome, as:

$Z=\prod_{t=1}^{\infty} C_{t}$ (S1)

In Equation S1, *Z* is cascade length, *t* is the time step, *t* = 1 is the time of the first fitness revision of the cascade, and the *C_t_* are Bernoulli RVs modified to have support on {1, [1 + 1/*t*]}. By definition, these cause size distributions are correlated strongly such that all causes following the first unity-valued cause are also unity with probability one, and causes previous to those are a nonlinear function of the preceding cause (i.e., *Z* grows by unity at each step until the first instance of *C_t_* = 1, at which point growth stops). The resulting cascade size distribution (for critical value 0.667) is shown in Figure A, panel (a). The discrete Weibull is consistent with this generalized model, providing natural fall-off at the limits of scaling. Of note, this result is obtained even though in this (original) model the cause size distributions increase “monotonically,” i.e. the cause size [1 + 1/t] is more probable than the alternative (unity).

**Figure A. Simulated distributions of complex system outcomes and fitted discrete Weibull:** (a) Preferential attachment model. [Conditions: 1000 vertices, final average degree 9.606, 4803 lines, 60% preferential attachment, 40% uniform attachment]; (b) Self-organized critical model. [Conditions: critical value 0.667]

The preferential attachment (PA) network model of Barabasi and Albert [3] is another general complex system model that may be viewed as autocorrelated first order growth. The original model of worldwide web network connections is constructed by adding a new vertex at each time step, connected by a constant number, *m*, of new edges to existing vertices with probability *k_i_*/Σ*k_i_*, in which *k_i_* is the degree of existing vertex *i* and Σ*k_i_* is the total number of edges in the network. Many real networks acquire edges in such a preferential, or autocorrelated, fashion [4]. The authors showed by simulation that the time-averaged rate at which vertices acquire edges is $\partial k_{i}/\partial t=k_{i}/2t$, in which *t* is the time step, giving *k_i_* = *m*(*t*/*t_i_*)^0.5^, in which *t_i_* is the time at which vertex *i* was added to the network. Thus, the process is first-order with respect to *k_i_*, and correlated in time. At any time, *t*, the distribution of *t_i_* across the network is rectangular on [0, *t*]. Therefore, to the extent that the empirical relationship between *k_i_* and *t_i_* holds, a right truncated power law distribution of *k_i_*, proportional to *k_i_^3^*, can be found over the range *t* = 0 – 1*,* by transformation of variables.

Alternatively, for this discrete process, the degree of vertex *i* at discrete time *t* can be written similarly to Equation S1 as a product, as follows:

$\begin{matrix} \Delta k_{i}=\frac{k_{i}}{2t}\Delta t \\ \left.k_{i}\right|_{t_{i}}=m \\ \left.k_{i}\right|_{t_{i}+1}=m+\frac{\left.k_{i}\right|_{t_{i}}}{2t} \\ =m\left[1+\frac{1}{2(t_{i}+1)}\right] \\ \left.k_{i}\right|_{t_{i}+2}=\left.k_{i}\right|_{t_{i}+1}+\frac{\left.k_{i}\right|_{t_{i}+1}}{2(t_{i}+2)} \\ =m\left[1+\frac{1}{2(t_{i}+1)}\right]\left[1+\frac{1}{2(t_{i}+2)}\right] \\ \left.k_{i}\right|_{t}=m\prod_{\tau=t_{i}+ 1}^{t} \left(1+\frac{1}{2\tau}\right) \end{matrix}$ (S2)

Thus the PA model can be viewed similarly to the SOC model as an autocorrelated first order model, specifically the product of random cause sizes from *τ* = 0 to *t*, each having support on {1, [1 + 1/(2*τ*)]}, with all cause sizes following the first [1 + 1/(2*τ*)]-valued size taking the same [1 + 1/(2*τ*)]-value with probability one. However, many real networks have a mixture of preferential and uniform attachment [5]. In Figure A, panel (b), the degree-distribution generated by a generalized model^(5)^, 60% preferential/40% uniform (1000 vertices, average degree 10) is shown together with a discrete Weibull. The discrete Weibull corresponds well with this generalized model.

**References**

1. BAK P, TANG C, WIESENFELD K. Self-Organized Criticality - an Explanation of 1/f Noise. Phys. Rev. Lett. 1987;59(4): 381-4.

2. Englehardt J, Swartout J. Predictive bayesian microbial dose-response assessment based on suggested self-organization in primary illness response: Cryptosporidium parvum. Risk Analysis 2006;26(2): 543-54.

3. Barabasi A, Albert R. Emergence of scaling in random networks RID E-2195-2011. Science 1999;286(5439): 509-12.

4. Krapivsky P, Redner S. Organization of growing random networks. Physical Review E 2001;63(6): 066123.

5. Pennock D, Flake G, Lawrence S, Glover E, Giles C. Winners don't take all: Characterizing the competition for links on the web. Proc. Natl. Acad. Sci. U. S. A. 2002;99(8): 5207-11.
