# Supplementary material for: Distributions of Autocorrelated First-Order Kinetic Outcomes: Illness Severity
Source: PLoS One. 2015 Jun 10;10(6):e0129042. doi: 10.1371/journal.pone.0129042 (PMC4465627; doi:10.1371/journal.pone.0129042)
Supplement: S2 File — (DOCX) [file pone.0129042.s002.docx]

**Log-log Linearity of Weibull Distributions with Variable *η***

It should be noted that when the parameter, *η*, of the Weibull distribution varies, the resulting mixture of asymptotic Weibull distributions elongates further, often appearing as a pure power law. Such data may either (a) be pooled across separate causal systems (e.g., natural disasters in total, or oil spills worldwide), or (b) represent single systems with varying degrees of first-order compounding, extents of autocorrelation among compounding increments, and/or degrees to which process increments are distributed exponential. For example, when the Weibull parameter, *η*, is distributed exponential (*θ*), the distribution of outcomes sizes can be found as $\mathrm{f}(z)=\int_{0}^{\infty} \frac{\eta}{\lambda} \left(\frac{z}{\lambda}\right)^{\eta-1}\exp\left(\frac{z}{\lambda}\right)^{\eta}\theta\exp(-\theta\eta) d\eta= \theta/[z(z/\lambda+\theta)^{2}]$, *z* ≥ 0, by collecting terms and constants within the integral to form a PDF that integrates to unity. The result is a truncated bifurcated power law with slope *m* = -1 at small *z*/*λ* and *m* = -3 otherwise. As another example, in Figure A, the distribution obtained by superposition of 30 Weibull distributions, $\mathrm{f}(x)=\sum_{i=1}^{30} \mathrm{f}_{i}(x)/30$f_i_(*x*), each having unit mean but varying *η* = 1, 1/2, … 1/30, is shown to be visually linear. Likewise, distributions of the sizes of fire and explosion catastrophes both in the U.S. and worldwide follow approximate power laws [1], and these distributions may be interpreted as mixtures of Weibull distributions with values of *η* that vary across these places and systems (due in turn to variations in the extent of first-order compounding, autocorrelation of process increments, and exponential character of process increments).

**Figure A. Weibull distributions with unit mean.** Values of parameter *η* correspond to 1 (--) and 30 (- -) causes, and the mixture distribution of the pooled outcomes of 30 networks with *η* corresponding to 1, 2, …, 30 causes (—).

**References**

1. Copolla A, Hall R. A risk comparison. NUREG/CR-1916, BNL-NUREG-51338, U.S. Nuclear Regulatory Commission, Washington, DC 1981.
