# Supplementary material for: Distributions of Autocorrelated First-Order Kinetic Outcomes: Illness Severity
Source: PLoS One. 2015 Jun 10;10(6):e0129042. doi: 10.1371/journal.pone.0129042 (PMC4465627; doi:10.1371/journal.pone.0129042)
Supplement: S1 Table — (DOCX) [file pone.0129042.s003.docx]

S1 Table. Data of Kjellström et al. [40,41], Sorted by Hair Mercury Level, on Childrens’ Test Scores Found Sensitive to Prenatal Mercury Exposure.

| ID no. | Hair Hg | TOLD-SL | WISC-RP | WISC-RF | MCC-PP | MCC-MS |
| --- | --- | --- | --- | --- | --- | --- |
| 59 | 0.5 | 81 | 84 | 87 | 44 | 56 |
| 299 | 0.7 | 116 | 123 | 127 | 66 | 64 |
| 153 | 0.78 | 58 | 77 | 72 | 40 | 46 |
| 219 | 0.78 | 85 | 100 | 92 | 64 | 56 |
| 349 | 0.79 | 113 | 106 | 114 | 64 | 66 |
| 44 | 0.8 | 92 | 121 | 116 | 66 | 66 |
| 244 | 0.83 | 70 | 96 | 86 | 72 | 68 |
| 28 | 0.9 | 71 | 126 | 109 | 59 | 69 |
| 279 | 0.93 | 81 | 102 | 100 | 67 | 67 |
| 168 | 0.94 | 66 | 75 | 78 | 42 | 47 |
| 69 | 0.99 | 130 | 129 | 129 | 70 | 58 |
| 169 | 1 | 58 | 81 | 71 | 43 | 52 |
| 204 | 1 | 74 | 96 | 84 | 59 | 73 |
| 209 | 1 | 79 | 104 | 101 | 42 | 56 |
| 264 | 1 | 93 | 121 | 116 | 68 | 56 |
| 304 | 1.01 | 50 | 48 | 46 | 22 | 22 |
| 124 | 1.03 | 68 | 120 | 107 | 69 | 76 |
| 54 | 1.08 | 96 | 117 | 109 | 70 | 69 |
| 38 | 1.09 | 67 | 77 | 84 | 50 | 56 |
| 43 | 1.1 | 86 | 112 | 112 | 72 | 74 |
| 354 | 1.1 | 67 | 92 | 86 | 62 | 56 |
| 268 | 1.12 | 70 | 98 | 90 | 46 | 58 |
| 239 | 1.13 | 62 | 97 | 91 | 60 | 66 |
| 74 | 1.16 | 77 | 108 | 106 | 68 | 68 |
| 114 | 1.16 | 63 | 88 | 84 | 41 | 42 |
| 223 | 1.18 | 89 | 117 | 112 | 73 | 74 |
| 278 | 1.3 | 66 | 82 | 71 | 51 | 57 |
| 9 | 1.31 | 108 | 105 | 109 | 68 | 75 |
| 134 | 1.32 | 73 | 92 | 87 | 62 | 66 |
| 358 | 1.32 | 104 | 106 | 106 | 64 | 75 |
| 263 | 1.33 | 90 | 100 | 102 | 71 | 71 |
| 364 | 1.34 | 93 | 96 | 94 | 53 | 62 |
| 89 | 1.37 | 74 | 84 | 81 | 57 | 67 |
| 243 | 1.39 | 86 | 106 | 102 | 53 | 71 |
| 178 | 1.4 | 68 | 109 | 96 | 67 | 69 |
| 189 | 1.4 | 93 | 105 | 100 | 59 | 52 |
| 269 | 1.4 | 70 | 95 | 96 | 66 | 66 |
| 319 | 1.41 | 66 | 84 | 83 | 48 | 63 |
| 129 | 1.44 | 89 | 108 | 109 | 64 | 63 |
| 68 | 1.45 | 74 | 75 | 71 | 42 | 47 |
| 369 | 1.46 | 82 | 115 | 108 | 72 | 60 |
| 199 | 1.47 | 59 | 86 | 76 | 52 | 58 |
| 48 | 1.48 | 51 | 98 | 89 | 68 | 65 |
| 148 | 1.48 | 68 | 98 | 94 | 66 | 75 |
| 284 | 1.48 | 98 | 128 | 121 | 72 | 62 |
| 339 | 1.5 | 85 | 100 | 92 | 50 | 58 |
| 359 | 1.5 | 89 | 100 | 100 | 75 | 77 |
| 18 | 1.52 | 83 | 105 | 101 | 63 | 67 |
| 194 | 1.55 | 70 | 93 | 92 | 49 | 58 |
| 19 | 1.58 | 94 | 106 | 101 | 66 | 64 |
| 159 | 1.58 | 94 | 124 | 121 | 61 | 78 |
| 183 | 1.58 | 50 | 81 | 68 | 37 | 62 |
| 208 | 1.58 | 83 | 96 | 92 | 59 | 56 |
| 29 | 1.6 | 104 | 117 | 110 | 72 | 66 |
| 149 | 1.64 | 58 | 90 | 83 | 42 | 46 |
| 318 | 1.65 | 64 | 92 | 85 | 53 | 55 |
| 34 | 1.66 | 59 | 78 | 77 | 54 | 55 |
| 154 | 1.66 | 64 | 98 | 88 | 58 | 75 |
| 3 | 1.67 | 62 | 95 | 88 | 72 | 56 |
| 113 | 1.67 | 64 | 82 | 81 | 46 | 58 |
| 228 | 1.71 | 89 | 128 | 120 | 61 | 73 |
| 308 | 1.74 | 63 | 74 | 76 | 58 | 74 |
| 179 | 1.78 | 85 | 104 | 101 | 53 | 56 |
| 49 | 1.8 | 88 | 126 | 123 | 73 | 67 |
| 99 | 1.8 | 52 | 88 | 87 | 50 | 51 |
| 188 | 1.8 | 82 | 92 | 98 | 57 | 38 |
| 94 | 1.83 | 82 | 112 | 106 | 53 | 63 |
| 298 | 1.84 | 63 | 82 | 74 | 53 | 59 |
| 368 | 1.84 | 94 | 71 | 62 | 42 | 58 |
| 258 | 1.85 | 111 | 114 | 122 | 61 | 71 |
| 108 | 1.86 | 55 | 92 | 84 | 48 | 59 |
| 64 | 1.9 | 73 | 73 | 74 | 42 | 55 |
| 173 | 1.91 | 82 | 82 | 85 | 50 | 62 |
| 343 | 1.93 | 71 | 104 | 92 | 52 | 61 |
| 14 | 1.94 | 71 | 82 | 77 | 48 | 52 |
| 198 | 1.97 | 74 | 98 | 92 | 56 | 68 |
| 8 | 2 | 77 | 98 | 91 | 55 | 52 |
| 259 | 2.08 | 54 | 86 | 73 | 50 | 62 |
| 214 | 2.1 | 101 | 104 | 107 | 68 | 77 |
| 93 | 2.11 | 78 | 104 | 111 | 71 | 73 |
| 353 | 2.14 | 66 | 85 | 81 | 64 | 67 |
| 33 | 2.15 | 75 | 98 | 86 | 63 | 68 |
| 363 | 2.2 | 62 | 85 | 80 | 64 | 78 |
| 288 | 2.22 | 78 | 85 | 80 | 65 | 73 |
| 88 | 2.26 | 81 | 108 | 102 | 59 | 67 |
| 338 | 2.26 | 81 | 85 | 84 | 58 | 75 |
| 309 | 2.29 | 67 | 93 | 87 | 73 | 63 |
| 203 | 2.34 | 64 | 84 | 76 | 66 | 71 |
| 184 | 2.39 | 50 | 98 | 84 | 47 | 45 |
| 229 | 2.4 | 77 | 112 | 99 | 69 | 66 |
| 128 | 2.43 | 58 | 72 | 68 | 47 | 69 |
| 233 | 2.44 | 78 | 87 | 90 | 58 | 54 |
| 39 | 2.51 | 107 | 117 | 123 | 64 | 56 |
| 123 | 2.54 | 89 | 96 | 97 | 66 | 63 |
| 174 | 2.55 | 81 | 100 | 96 | 59 | 67 |
| 13 | 2.56 | 73 | 80 | 78 | 41 | 46 |
| 4 | 2.6 | 111 | 130 | 132 | 72 | 62 |
| 63 | 2.6 | 74 | 86 | 89 | 50 | 55 |
| 79 | 2.61 | 59 | 56 | 75 | 52 | 62 |
| 119 | 2.62 | 50 | 45 | 40 | 22 | 22 |
| 283 | 2.68 | 92 | 108 | 109 | 57 | 62 |
| 289 | 2.69 | 73 | 106 | 92 | 50 | 65 |
| 213 | 2.7 | 83 | 115 | 100 | 52 | 56 |
| 238 | 2.72 | 68 | 109 | 91 | 70 | 65 |
| 78 | 2.74 | 71 | 114 | 99 | 56 | 66 |
| 303 | 2.78 | 75 | 108 | 105 | 59 | 69 |
| 109 | 2.8 | 81 | 128 | 111 | 61 | 77 |
| 133 | 2.8 | 90 | 114 | 106 | 57 | 61 |
| 158 | 2.8 | 66 | 102 | 92 | 63 | 57 |
| 193 | 2.8 | 74 | 100 | 88 | 59 | 62 |
| 98 | 2.82 | 60 | 111 | 95 | 65 | 71 |
| 234 | 2.86 | 78 | 102 | 98 | 56 | 67 |
| 118 | 2.87 | 68 | 115 | 105 | 59 | 62 |
| 218 | 2.87 | 68 | 88 | 86 | 40 | 54 |
| 53 | 2.9 | 74 | 96 | 91 | 52 | 66 |
| 58 | 2.9 | 78 | 72 | 69 | 46 | 54 |
| 348 | 2.96 | 78 | 114 | 111 | 66 | 67 |
| 73 | 2.99 | 88 | 100 | 90 | 53 | 58 |
| 92 | 3 | 77 | 104 | 96 | 54 | 66 |
| 297 | 3.02 | 92 | 120 | 110 | 69 | 65 |
| 157 | 3.04 | 79 | 101 | 98 | 51 | 58 |
| 167 | 3.07 | 79 | 100 | 93 | 50 | 56 |
| 237 | 3.11 | 56 | 69 | 65 | 39 | 62 |
| 12 | 3.12 | 50 | 45 | 40 | 22 | 22 |
| 227 | 3.15 | 107 | 100 | 105 | 60 | 66 |
| 57 | 3.16 | 109 | 132 | 133 | 67 | 50 |
| 342 | 3.16 | 60 | 96 | 77 | 56 | 65 |
| 307 | 3.19 | 89 | 96 | 102 | 49 | 58 |
| 2 | 3.2 | 89 | 118 | 106 | 58 | 66 |
| 37 | 3.2 | 62 | 96 | 86 | 47 | 45 |
| 187 | 3.2 | 103 | 113 | 118 | 75 | 67 |
| 177 | 3.28 | 85 | 114 | 112 | 67 | 78 |
| 357 | 3.28 | 70 | 102 | 90 | 60 | 65 |
| 302 | 3.3 | 66 | 88 | 78 | 42 | 62 |
| 317 | 3.3 | 79 | 96 | 91 | 57 | 52 |
| 337 | 3.39 | 73 | 105 | 102 | 67 | 64 |
| 127 | 3.42 | 82 | 95 | 91 | 47 | 66 |
| 242 | 3.5 | 62 | 96 | 89 | 55 | 63 |
| 267 | 3.59 | 54 | 80 | 72 | 44 | 42 |
| 152 | 3.68 | 73 | 84 | 77 | 48 | 66 |
| 207 | 3.7 | 90 | 109 | 110 | 53 | 64 |
| 132 | 3.74 | 73 | 86 | 81 | 46 | 50 |
| 282 | 3.75 | 85 | 95 | 93 | 57 | 65 |
| 67 | 3.77 | 77 | 102 | 99 | 64 | 53 |
| 77 | 3.8 | 75 | 100 | 92 | 61 | 78 |
| 62 | 3.87 | 83 | 108 | 99 | 69 | 78 |
| 122 | 3.94 | 54 | 95 | 89 | 62 | 59 |
| 97 | 4.04 | 89 | 111 | 109 | 68 | 68 |
| 47 | 4.06 | 71 | 100 | 89 | 68 | 73 |
| 172 | 4.22 | 79 | 118 | 103 | 64 | 74 |
| 352 | 4.23 | 74 | 118 | 100 | 63 | 68 |
| 192 | 4.3 | 66 | 82 | 81 | 49 | 62 |
| 197 | 4.56 | 64 | 95 | 92 | 54 | 59 |
| 277 | 4.57 | 88 | 111 | 105 | 61 | 68 |
| 362 | 4.58 | 58 |  |  |  |  |
| 27 | 4.6 | 85 | 95 | 105 | 52 | 52 |
| 287 | 4.6 | 66 | 82 | 85 | 53 | 50 |
| 107 | 4.61 | 59 | 98 | 94 | 55 | 63 |
| 17 | 4.64 | 103 | 128 | 118 | 69 | 69 |
| 147 | 4.68 | 63 | 75 | 68 | 40 | 57 |
| 212 | 4.68 | 68 | 101 | 96 | 58 | 62 |
| 262 | 4.74 | 74 | 118 | 110 | 71 | 78 |
| 7 | 4.77 | 74 | 102 | 97 | 69 | 71 |
| 182 | 4.9 | 93 | 105 | 101 | 61 | 63 |
| 232 | 4.92 | 90 | 112 | 100 | 64 | 66 |
| 32 | 4.93 | 79 | 86 | 88 | 50 | 65 |
| 217 | 5 | 71 | 95 | 95 | 66 | 73 |
| 202 | 5.05 | 70 | 86 | 84 | 54 | 56 |
| 87 | 5.19 | 88 | 73 | 87 | 47 | 60 |
| 112 | 5.24 | 86 | 81 | 90 | 36 | 44 |
| 257 | 5.34 | 67 | 88 | 89 | 62 | 78 |
| 117 | 5.41 | 62 | 68 | 69 | 42 | 52 |
| 347 | 5.47 | 93 | 120 | 114 | 67 | 68 |
| 52 | 5.65 | 82 | 96 | 96 | 66 | 75 |
| 312 | 5.69 | 74 | 96 | 90 | 59 | 58 |
| 42 | 5.92 | 109 | 100 | 111 | 55 | 52 |
| 72 | 5.97 | 83 | 109 | 105 | 58 | 65 |
| 366 | 6 | 82 | 101 | 97 | 60 | 70 |
| 361 | 6.12 | 62 | 104 | 98 | 51 | 53 |
| 356 | 6.14 | 86 | 98 | 91 | 54 | 65 |
| 351 | 6.2 | 74 | 88 | 86 | 53 | 55 |
| 346 | 6.23 | 73 | 101 | 103 | 49 | 43 |
| 341 | 6.3 | 51 | 70 | 54 | 22 | 27 |
| 336 | 6.33 | 50 | 69 | 68 | 47 | 71 |
| 316 | 6.52 | 64 | 128 | 105 | 58 | 62 |
| 301 | 6.6 | 62 | 90 | 78 | 58 | 70 |
| 306 | 6.6 | 104 | 126 | 123 | 71 | 63 |
| 311 | 6.6 | 60 | 77 | 92 | 57 | 69 |
| 296 | 6.61 | 71 | 85 | 84 | 64 | 58 |
| 286 | 6.68 | 70 | 102 | 87 | 47 | 67 |
| 281 | 6.8 | 79 | 87 | 83 | 41 | 56 |
| 276 | 6.83 | 56 | 98 | 91 | 52 | 56 |
| 261 | 7.14 | 67 | 117 | 109 | 71 | 71 |
| 266 | 7.14 | 59 | 88 | 72 | 54 | 53 |
| 256 | 7.15 | 79 | 80 | 83 | 56 | 63 |
| 241 | 7.28 | 63 | 90 | 91 | 50 | 78 |
| 236 | 7.3 | 85 | 101 | 101 | 52 | 60 |
| 231 | 7.36 | 74 | 104 | 97 | 64 | 69 |
| 221 | 7.4 | 77 | 100 | 93 | 56 | 46 |
| 226 | 7.4 | 79 | 106 | 102 | 61 | 67 |
| 206 | 7.5 | 89 | 90 | 81 | 50 | 58 |
| 211 | 7.5 | 89 | 105 | 96 | 51 | 53 |
| 216 | 7.5 | 64 | 93 | 89 | 64 | 64 |
| 201 | 7.7 | 68 | 93 | 88 | 51 | 47 |
| 196 | 7.71 | 77 | 105 | 100 | 69 | 69 |
| 191 | 7.78 | 86 | 91 | 87 | 50 | 50 |
| 186 | 7.93 | 77 | 104 | 99 | 54 | 45 |
| 181 | 7.96 | 67 | 95 | 88 | 48 | 64 |
| 176 | 7.99 | 50 | 69 | 59 | 35 | 52 |
| 171 | 8.01 | 64 | 88 | 84 | 58 | 67 |
| 166 | 8.02 | 73 | 87 | 79 | 47 | 41 |
| 156 | 8.2 | 108 | 98 | 100 | 49 | 62 |
| 151 | 8.46 | 56 | 98 | 84 | 53 | 54 |
| 146 | 8.5 | 81 | 105 | 100 | 61 | 65 |
| 131 | 8.6 | 78 | 95 | 95 | 54 | 60 |
| 126 | 8.61 | 71 | 104 | 91 | 55 | 54 |
| 121 | 8.76 | 66 | 102 | 87 | 60 | 51 |
| 116 | 8.84 | 63 | 100 | 95 | 60 | 76 |
| 106 | 9.42 | 60 | 87 | 75 | 42 | 60 |
| 111 | 9.42 | 98 | 106 | 113 | 43 | 46 |
| 96 | 9.55 | 85 | 102 | 103 | 50 | 62 |
| 91 | 9.7 | 90 | 109 | 108 | 64 | 54 |
| 86 | 9.75 | 62 | 104 | 88 | 54 | 60 |
| 76 | 10 | 66 | 95 | 89 | 60 | 68 |
| 71 | 10.12 | 105 | 121 | 116 | 74 | 70 |
| 66 | 10.66 | 81 | 109 | 92 | 55 | 61 |
| 61 | 10.7 | 78 | 96 | 100 | 68 | 69 |
| 56 | 11.8 | 127 | 92 | 102 | 46 | 63 |
| 51 | 12 | 77 | 121 | 114 | 68 | 67 |
| 46 | 12.1 | 55 | 71 | 68 | 44 | 54 |
| 41 | 12.16 | 74 | 115 | 109 | 62 | 54 |
| 36 | 12.87 | 52 | 73 | 76 | 49 | 54 |
| 31 | 13.52 | 54 | 101 | 86 | 61 | 78 |
| 26 | 14.72 | 50 | 74 | 70 | 31 | 40 |
| 16 | 16.82 | 64 | 75 | 72 | 36 | 38 |
| 11 | 17.58 | 77 | 74 | 79 | 47 | 58 |
| 6 | 19.58 | 59 | 91 | 84 | 56 | 65 |
| 1 | 86.4 | 74 | 100 | 90 | 54 | 58 |
