# Supplementary material for: Distributions of Autocorrelated First-Order Kinetic Outcomes: Illness Severity
Source: PLoS One. 2015 Jun 10;10(6):e0129042. doi: 10.1371/journal.pone.0129042 (PMC4465627; doi:10.1371/journal.pone.0129042)
Supplement: S2 Table — (DOCX) [file pone.0129042.s004.docx]

S2 Table. Time-to-Tumor Data of the ED_01_ Study: Bladder Neoplasms, Liver Neoplasms, and Bladder Carcinomas [45].

|  | **Bladder Neoplasms**  (No. bladder neoplasms/No. dead, moribund, and sacrificed mice examined) | | | | | | | | | | |
| --- | --- | --- | --- | --- | --- | --- | --- | --- | --- | --- | --- |
| **Months On Study** | **Dose (ppm)** | | | | | | | | | | |
|  | **0** | | **30** | **35** | **45** | | **60** | | **75** | **100** | **150** |
| 9 | 3/204 | | 2/164 | 1/92 | 1/65 | | 0/357 | | 0/193 | 1/171 | 2/172 |
| 12 | 1/164 | | 1/52 | 0/27 | 0/14 | | 0/283 | | 1/153 | 0/149 | 11/152 |
| 14 | 0/133 | | 1/42 | 0/24 | 0/14 | | 0/239 | | 0/124 | 0/128 | 23/126 |
| 15 | 1/114 | | 0/75 | 0/34 | 3/20 | | 0/204 | | 1/109 | 0/98 | 32/100 |
| 16 | 1/204 | | 1/64 | 0/59 | 1/301 | | 0/288 | | 1/194 | 2/100 | 42/109 |
| 17 | 0/152 | | 1/69 | 2/443 | 3/300 | | 0/229 | | 1/166 | 6/85 | 45/82 |
| 18 | 2/553 | | 14/2008 | 4/1103 | 6/545 | | 5/410 | | 4/382 | 10/213 | 107/207 |
| 24 | 2/759 | | 9/2105 | 5/1357 | 4/881 | | 6/756 | | 13/586 | 51/297 | 236/313 |
| 33 | 1/101 | | 5/443 | 0/200 | 2/103 | | 2/66 | | 12/75 | 21/31 | 11/11 |
|  | **Liver Neoplasms**  (No. liver neoplasms/No. dead, moribund, and sacrificed mice examined) | | | | | | | | | | |
| **Months On Study** | **Dose (ppm)** | | | | | | | | | | |
|  | **0** | **30** | | **35** | | **45** | | **60** | **75** | **100** | **150** |
| 9 | 0/199 | 1/147 | | 1/76 | | 0/52 | | 0/345 | 0/186 | 1/168 | 1/169 |
| 12 | 0/164 | 1/151 | | 2/27 | | 1/14 | | 2/283 | 0/153 | 3/149 | 2/152 |
| 14 | 1/133 | 1/42 | | 0/25 | | 2/14 | | 1/243 | 0/124 | 1/127 | 1/127 |
| 15 | 0/115 | 1/75 | | 1/35 | | 0/20 | | 3/203 | 1/109 | 5/99 | 1/100 |
| 16 | 1/205 | 2/66 | | 2/61 | | 3/304 | | 6/287 | 7/193 | 2/100 | 7/110 |
| 17 | 0/153 | 4/69 | | 5/443 | | 6/302 | | 8/230 | 9/166 | 3/85 | 1/82 |
| 18 | 6/555 | 34/2014 | | 20/1102 | | 15/550 | | 13/411 | 17/382 | 19/213 | 24/211 |
| 24 | 20/762 | 164/2109 | | 128/1361 | | 98/888 | | 118/758 | 118/587 | 76/297 | 126/314 |
| 33 | 17/100 | 135/445 | | 72/100 | | 42/103 | | 30/67 | 37/75 | 22/31 | 9/11 |
|  | **Bladder Carcinomas**  (No. mice with bladder carcinomas/No. Dead, Moribund, and Sacrificed Mice Examined) | | | | | | | | | | |
| **Months On Study** | **Dose (ppm)** | | | | | | | | | | |
|  | **0** | | **30** | **35** | **45** | | **60** | | **75** | **100** | **150** |
| 9 | 3/204 | | 2/164 | 1/92 | 1/65 | | 0/357 | | 0/193 | 1/171 | 2/172 |
| 12 | 1/164 | | 1/52 | 1/27 | 0/14 | | 0/283 | | 0/153 | 1/149 | 0/152 |
| 14 | 0/133 | | 1/42 | 0/24 | 0/14 | | 0/239 | | 0/124 | 0/128 | 23/126 |
| 15 | 1/114 | | 0/75 | 0/34 | 3/20 | | 0/204 | | 1/109 | 0/98 | 32/100 |
| 16 | 1/204 | | 1/64 | 0/59 | 1/301 | | 0/288 | | 1/194 | 2/100 | 42/109 |
| 17 | 0/152 | | 1/69 | 2/443 | 3/300 | | 0/229 | | 1/166 | 6/85 | 45/82 |
| 18 | 2/553 | | 14/2008 | 4/1103 | 6/545 | | 5/410 | | 4/382 | 10/213 | 107/207 |
| 24 | 2/759 | | 9/2105 | 5/1357 | 4/881 | | 6/756 | | 13/586 | 51/297 | 236/313 |
| 33 | 1/101 | | 5/443 | 0/200 | 2/103 | | 2/66 | | 12/75 | 21/31 | 11/11 |
| Total | 11/2383 | | 34/5022 | 12/3339 | 20/2243 | | 13/2832 | | 33/1982 | 91/1272 | 509/1272 |
